# Reactive oxygen species and nitric oxide imbalances lead to *in vivo* and *in vitro* arrhythmogenic phenotype in acute phase of experimental Chagas disease

**DOI:** 10.1371/journal.ppat.1008379

**Published:** 2020-03-11

**Authors:** Artur Santos-Miranda, Julliane Vasconcelos Joviano-Santos, Grazielle Alves Ribeiro, Ana Flávia M. Botelho, Peter Rocha, Leda Quercia Vieira, Jader Santos Cruz, Danilo Roman-Campos

**Affiliations:** 1 Department of Biochemistry and Immunology, Instituto de Ciências Biológicas, Universidade Federal de Minas Gerais, Belo Horizonte, Minas Gerais, Brazil; 2 Department of Biophysics, Universidade Federal de São Paulo, São Paulo, Brazil; 3 Department of Veterinary Medicine, Escola de Veterinária e Zootecnia, Universidade Federal de Goiás, Goiânia, Brazil; Universidade Federal do Rio de Janeiro, BRAZIL

## Abstract

Chagas Disease (CD) is one of the leading causes of heart failure and sudden death in Latin America. Treatments with antioxidants have provided promising alternatives to ameliorate CD. However, the specific roles of major reactive oxygen species (ROS) sources, including NADPH-oxidase 2 (NOX2), mitochondrial-derived ROS and nitric oxide (NO) in the progression or resolution of CD are yet to be elucidated. We used C57BL/6 (WT) and a gp91^PHOX^ knockout mice (PHOX^-/-^), lacking functional NOX2, to investigate the effects of ablation of NOX2-derived ROS production on the outcome of acute chagasic cardiomyopathy. Infected PHOX^-/-^ cardiomyocytes displayed an overall pro-arrhythmic phenotype, notably with higher arrhythmia incidence on ECG that was followed by higher number of early afterdepolarizations (EAD) and 2.5-fold increase in action potential (AP) duration alternans, compared to AP from infected WT mice. Furthermore, infected PHOX^-/-^ cardiomyocytes display increased diastolic [Ca^2+^], aberrant Ca^2+^ transient and reduced Ca^2+^ transient amplitude. Cardiomyocyte contraction is reduced in infected WT and PHOX^-/-^ mice, to a similar extent. Nevertheless, only infected PHOX^-/-^ isolated cardiomyocytes displayed significant increase in non-triggered extra contractions (appearing in ~75% of cells). Electro-mechanical remodeling of infected PHOX^-/—^cardiomyocytes is associated with increase in NO and mitochondria-derived ROS production. Notably, EADs, AP duration alternans and *in vivo* arrhythmias were reverted by pre-incubation with nitric oxide synthase inhibitor L-NAME. Overall our data show for the first time that lack of NOX2-derived ROS promoted a pro-arrhythmic phenotype in the heart, in which the crosstalk between ROS and NO could play an important role in regulating cardiomyocyte electro-mechanical function during acute CD. Future studies designed to evaluate the potential role of NOX2-derived ROS in the chronic phase of CD could open new and more specific therapeutic strategies to treat CD and prevent deaths due to heart complications.

## Author summary

Chagas disease (CD) is an important neglected disease mainly found in developing countries. However, due to migration flow, it became a health problem worldwide. Infection by *Trypanosoma cruzi* typically occurs after an infected Triatominae vector takes a blood meal and leaves parasites in its feces nearby the bite wound. Two distinct symptomatic stages of CD are typical, an acute phase that lasts few weeks and a chronic stage, that can last for 10–30 years after infection, and may lead to severe cardiac arrhythmias and death. Here, we used an experimental model of acute CD to study the involvement of reactive oxygen species (ROS) and nitric oxide (NO) with structural and functional remodeling of cardiomyocytes and the heart. We found that an unbalanced production of two sources of reactive species may worsen cellular electromechanical dysfunction, leading to more severe arrhythmias despite additional structural changes in heart tissue. Interestingly, it was possible to attenuate the unbalanced relationship between ROS and NO by inhibition of NO production, leading to recovery of electrical function of heart cells and ameliorating *in vivo* heart arrhythmia. Thus, in the long term, our study may contribute to the development of new therapies to individuals with the cardiac form of CD.

## Introduction

Chagas disease (CD) is a parasitic disease caused by the protozoan *Trypanosoma cruzi* [1]. It is estimated that 6 million people are afflicted by the disease, mostly in South America [2]. However, migration has made the disease prevalent in other parts of the world [2,3]. In South America, CD is a leading cause of heart failure and sudden death [4]. Additionally, CD is considered one of the most onerous, neglected tropical diseases [5], with an estimated overall global cost of $7.19 billion/year. Surprisingly, more than 10% of these costs arise from the United States and Canada, non-endemic regions [6]. Transmission may happen by contact with fecal materials from blood-sucking triatominae bugs, blood or blood derivatives, by the congenital route, or by consuming food contaminated with infected insect components [2,7]. Among the several routes, oral transmission through contaminated food is currently the main source of transmission in Brazil [8] and has a more severe clinical course and higher mortality rate [9].

Chagasic cardiomyopathy is the most severe clinical form of CD. According to Prata [7], 20–30% of infected patients have heart diseases but no symptoms. Some of these patients, however, develop arrhythmias and/or heart failure.

During infection with *T*. *cruzi*, the host’s attempt to control parasite burden is associated with an elevation of reactive oxygen species (ROS) and nitric oxide (NO^.^, here only NO) levels that lead to oxidative stress in both acute and chronic phases of infection [10,11,12,13,14]. Oxidative stress in the heart is associated with matrix remodeling [15] as well as remodeling of cardiomyocytes electric and contractile function [16], and could be key factors to trigger heart dysfunction that occurs in CD. In line with this rationale, treatment with non-selective antioxidants have provided promising alternatives to prevent [17] and even to revert [18] heart dysfunction in experimental models of CD. In addition, new evidence suggests the involvement of ROS production as being important to parasite growth in the vertebrate host, apart from its role in parasite control [19]. Several sources of ROS and NO contribute to the net oxidative status of cardiomyocytes. However, the individual role of ROS generators in the pathogenesis of CD is yet to be elucidated, in order to control parasite burden and still keep the physiological signaling that ROS and NO exert in both electrical [20,21] and contractile [22] functions of cardiomyocytes. Therefore, targeting isolated sources of reactive chemical species provides a better understanding of the contribution of specific ROS in the progression or resolution of infection, as well as how the cardiac disease outcome is related to these specific components. In this study we used gp91^PHOX^ knockout mice (PHOX^-/-^) that lack the catalytic unit of NADPH-oxidase 2 (NOX2) as model [23,24]. NOX2 is a key source of ROS in cardiomyocytes, since NOX2-derived ROS can further induce activation of other ROS generators [15]. Furthermore, NOX2 is considered the main source of ROS during infection [25]. We hypothesized that ablation of NOX2-derived ROS could modulate the outcome of cardiac dysfunction during the acute phase of experimental CD.

In this study, PHOX^-/-^ and WT C57BL/6 mice were used in our experimental infection with *T*. *cruzi*, to investigate: 1) the influence of ablation of NOX2-derived ROS on the outcome of acute infection with *T*. *cruzi*. 2) The cellular basis underlying the modulation of the heart electrical properties during acute infection with *T*. *cruzi*. 3) The role of ROS and NO levels and their interplay to the outcome of acute infection with *T*. *cruzi*. Our results contribute to the overall knowledge of the pathogenesis of CD and might help to delineate more specific and accurate approaches to treat this debilitating disease.

## Methods

### Experimental models

We used male C57BL/6 wild-type (WT) and gp91^PHOX^-deficient (PHOX^-/-^) [23] mice in the C57BL/6 background, 8–12 weeks-old. WT mice were obtained from CEBIO (ICB, UFMG, Belo Horizonte, MG, Brazil). PHOX^-/-^ mice were originally obtained from Jackson Laboratories (Bar Harbor, Maine, USA) and bred in the Gnotobiology and Immunology Laboratory (UFMG, Belo Horizonte, MG, Brazil). Experiments were performed 15 ± 1 days post infection (d.p.i).

### Ethics statement

All mouse-related procedures were previously approved by the Institutional Animal Care and Use Committee at Universidade Federal de Minas Gerais (UFMG) (protocol #214/2011). All experiments were conducted according to Animal Research: Reporting *in Vivo* Experiments (ARRIVE).

### Infection

The Y strain of *T*. *cruzi* was used in all experiments. Trypomastigotes were maintained by blood passage in Swiss mice every 7 days. Trypomastigotes were obtained from heparinized blood, counted and used for infection. WT and PHOX^-/-^ mice were injected in the peritoneal cavity with 10^3^ trypomastigotes. Parasitemia was assessed by counting trypomastigotes in 5 μL of tail vein blood every day from the 3^rd^ day post-infection until 13^th^ day post-infection. The number of parasites per mL was calculated as previously described [26]. Mortality of infected mice was monitored daily. Treatment with nitric oxide synthases (NOS) inhibitor N(ω)-nitro-l-arginine methyl ester (l-NAME at 2 mM) was performed by oral administration added to the drinking water, during 15 days after infection. l-NAME solution was changed every day.

### Macrophage ROS detection assay

The macrophages used in this study were isolated from the peritoneal cavity of mice 4 days after injection of 2 mL of 3% thioglycollate medium (BD, Le Pont de Claix, France) into the peritoneal cavity. After this time, mice were euthanized and the peritoneum cells were harvested by repeated cycles of aspiration and re-injection with 10 mL of cold PBS in 10 mL syringe with a 24 G needle. More than 80% of the cells harvested were macrophages. Freshly obtained cells were centrifuged at 4 °C, 1,500 *g* for 10 min, and resuspended in RPMI medium without phenol red containing 10% fetal bovine serum (Cultilab, Campinas, SP, Brazil), 2 mM l-glutamine (Sigma-Aldrich, St. Louis, MO, USA), 100 u/mL penicillin and 100 μg/mL streptomycin (Gibco BRL Life Technologies, Grand Island, NY, USA). Macrophages (1 x 10^6^ cells/well) were plated in 96 well opaque plates (NUNC, Rochester, NY, USA). After 2 h, 0.05 mM luminol (5-amino-2,3-dihydro-1,4-phthalazinedione; Sigma-Aldrich) and *T*. *cruzi* trypomastigotes (10 parasites per macrophage) or zymosan (1 x 10^7^ U per well) were added to each well. Measurements were performed for 120 minutes at two-minute intervals. Production of ROS was assayed by the light intensity generated by the reaction between ROS and luminol and expressed as relative light units (RLU).

### Electrocardiography

Electrocardiographic (ECG) recordings were performed using a six-channel non-invasive electrocardiograph (ECG-PC version 2.07, Brazilian Electronic Technology—TEB, Belo Horizonte, MG, Brazil), for 5 minutes. Mice were anesthetized with a combination of xylazine (12.5 mg/kg) and ketamine (87.5 mg/kg). Readings were made in DII, at 50 mm/s, and 2 N.

### Histological assessment of cardiac morphology and fibrosis

For histological analysis, mice (n = 4 animals per group) were euthanatized and hearts were collected. Cardiac tissues were immersed in 4% paraformaldehyde in 0.1 M phosphate buffer, pH 7.4, for 24 h at 4 °C. The tissues were dehydrated by sequential washes with 70%, 80%, 90%, and 100% ethanol and embedded in paraffin. Transversal sections (5 μm) were collected using a microtome (model HM335E; Microm, Inc., Minneapolis, MN, USA), starting from the basal area of the heart and then, stained with Hematoxylin-eosin for general cell histology and morphometry. Specifically, for histological assessment of cardiac fibrosis, transversal sections were also stained with Masson’s trichrome. Tissue sections (3 for each animal) were examined with an inverted microscope (Zeiss Axiovert 100M), and analyzed using the ImageJ software (NIH, Bethesda, Maryland,USA) using similar method as previously published. Morphometric analysis was accessed using a pre-defined grid and a cell counter tool for measuring, set with 1.000 grid intersection points per mice, from 20 frames (S1 Fig).

### Cardiomyocyte isolation

Freshly isolated left ventricle cardiomyocytes were obtained following a previously described method [27], with minor modifications. After isolation, cardiomyocytes were kept in Tyrode solution (containing, in mM: 140 NaCl, 5.4 KCl, 0.5 MgCl_2_, 0.33 NaH_2_PO_4_, 11.0 glucose, 5.0 HEPES, and 1.8 CaCl_2_, pH 7.4) at room temperature (22–25 °C). Experiments were conducted for at most 4 h after cell isolation.

### Cellular electrophysiology

Whole-cell patch clamp recordings were obtained using an EPC-10 patch clamp amplifier (HEKA, Holliston, Massachusetts, USA) at room temperature (22–25 °C), in the voltage-clamp mode for current recordings and current-clamp mode for action potential (AP) recordings [28]. Glass pipettes were pulled with 1–2 MΩ tip resistance and cells with series resistance superior to 8 MΩ were not considered in the analysis, in order to prevent significant voltage-clamp errors. Furthermore, all current measurements were electronically compensated for series resistance (60–70%). In all records, cells were bathed with Tyrode solution. After achieving whole-cell configuration, cells were kept resting for 2–3 minutes, in order to allow proper equilibration between pipette solution and intracellular media. During action potential (AP) recordings, pipettes were filled with an internal solution composed of, in mM: 20 KCl; 130 aspartic acid; 130 KOH; 10 HEPES; 2 MgCl_2_; 5 NaCl, pH 7.2. After establishment of whole-cell configuration, recording mode was immediately switched to current-clamp mode and resting membrane potential was measured. AP recordings were sampled at 10 kHz. For L-type calcium current (I_Ca-L_) measurements, pipettes were filled with a solution composed of, in mM: 120 CsCl, 20 TEA-Cl, 5 NaCl, 10 HEPES, 5 EGTA, pH 7.2. During outward potassium current (I_K_) measurements, pipettes were filled with a solution containing, in mM: 140 KCl, 1 MgCl_2_, 10 EGTA, 10 HEPES, 5 glucose, pH 7.2. During I_K_ measurements, 0.1 mM Cd^2+^ was added to external Tyrode solution in order to block calcium currents. In all voltage-clamp protocols, a pre-pulse of 50 ms duration, from holding potential to -40 mV was applied to inactivate sodium current. Current records were sampled at 10 kHz and 5 kHz, respectively, for I_Ca-L_ and I_K_. I_Ca-L_ and I_K_ current densities were plotted against several tested membrane potentials and fitted with a Boltzmann equation in the form:

$$I_{\left(V_{m}\right)}=G_{\text{max}}*\frac{\left(V_{m}-E_{i}\right)}{\left(1-e^{\left(\frac{\left(V_{m}-V_{0.5}\right)}{s}\right)}\right)},$$

where *G*_*max*_ is the maximal conductance; *V*_*m*_ is the tested membrane potential; *E*_*i*_, is the calculated electrochemical equilibrium potential for ion *i*; *V*_*0*.*5*_ is the membrane potential where 50% of the channels are activated and *S* is the slope factor.

### Sarcomere contraction measurements

Cardiomyocyte sarcomere contraction properties were evaluated using a high speed NTSC camera (MyoCamCCD100V, Ionoptix, Milton, MA, USA) through a fast Fourier transform for sarcomere deconvolution-based analysis (IonWizard, Ionoptix, Milton, MA, USA). During this experiments, freshly isolated left ventricle cardiomyocytes were placed in a coverslip bathed with Tyrode solution and assembled into a chamber containing a pair of platinum electrodes from which cells were field stimulated (MyoPacer, IonOptix, Milton, MA, USA) with 4 ms duration and 60 V amplitude biphasic pulses, at stimulation frequency of 1 Hz. Signal was collected at 250 Hz rate. Five consecutive events of sarcomere shortening and re-lengthening were averaged for each cell analysis. The occurrence of extra contractions was evaluated through 60 s sarcomere detected contractions using the same stimulation protocol. The resting sarcomere length was measured using a fast Fourier transform algorithm (IonWizard, Ionoptix, Milton, MA, USA), in a relaxed state, without stimulation. All experiments were performed at room temperature (22–25 °C).

### Fluorescence measurements of intracellular cell calcium

Global intracellular calcium transients were elicited simultaneously with sarcomere contraction experiments, using the same stimulation protocol. Cardiomyocytes were loaded with 1 μM of the dual-excitation fluorescence probe Fura2-AM (Santa Cruz, California, USA) for 20 min, at room temperature and protected from light. Excitation was performed at 340/380 nm using a high-speed shutter (Hyper-Switch, IonOptix, Milton, MA, USA) and fluorescence emission was detected using photomultiplier tube, controlled and digitized by the fluorescence system interface (FSI700, IonOptix, Milton, MA, USA). The relation between fluorescence obtained from dual excitation was used to calculate calcium concentration according to the following equation: $\left[Ca^{2+}\right]=Kd*\frac{R-Rmin}{Rmax-R}*\frac{Sf2}{Sb2}$ [29], In which R_max_ and R_min_ are the ratio of fluorescence in depleted and saturated Ca^2+^ condition, obtained during *in vivo* calibration, Sf2 and Sb2 are the fluorescence arbitrary values for 380 nm excitation wavelength in depleted and saturated Ca^2+^ condition, and *K*_*d*_ is the Ca^2+^-Fura-2 dissociation constant (assumed as 225 nM). *In vivo* calibration was performed according to manufacturer instructions, using 5 μM ionomycin to modulate cell calcium concentration. Calibration after each Fura2-AM-load (5–6 cells) was averaged and used to calculate calcium concentration of cells from the same load, ensuring accuracy of measurements. Five consecutive events of global calcium transient were averaged for each cell analysis. Experiments were conducted at room temperature (22–25 °C).

### Confocal microscopy

Confocal imaging was performed using a Zeiss LSM 880 (Carl Zeiss, Germany) at UFMG image acquisition and processing center (CAPI–ICB–UFMG). Freshly isolated cardiomyocytes were loaded with respective probes: 4-amino-5-methylamino-2’,7’-difluorofluorescein diacetate for assessment of NO production (DAF-FM 5 μM, incubated for 30 minutes at room temperature); the indicator of mitochondrial superoxide anions production MitoSOX (5 μM, incubated for 15 minutes at 37 °C); Dihydroethidium for assessment of total superoxide anions production (DHE 5 μM, incubated for 30 minutes at 37 °C). All loadings were performed protected from light and under gentle stir. After loading, cells were centrifuged (1000 rpm for 30 s) and bathed in Tyrode solution. Optical slice was set to 2 μm in all recorded images, and ImageJ software was used for image processing and analysis. All experiments were conducted at room temperature.

### Statistical analysis

Data are presented as means ± standard error (SE), unless when indicated. Statistical significance of parametric data between groups was determined by ANOVA, followed by Tukey’s test after verification of normality distribution using Kolmogorov–Smirnov test. Data which failed to fit in a Gaussian distribution were compared using Kruskal-Wallis’ test followed by Dunns’s post test. Frequency distribution of EADs and extra-contractions events was tested with Fisher’s exact test. Significance was set at p < 0.05. Data were analyzed using GraphPad Prism (GraphPad Software, USA).

## Results

### Infected PHOX^-/-^ mice display high arrhythmogenic ECG that is prevented by NOS inhibition after acute infection with *T*. *cruzi* Y strain

**Fig 1:**
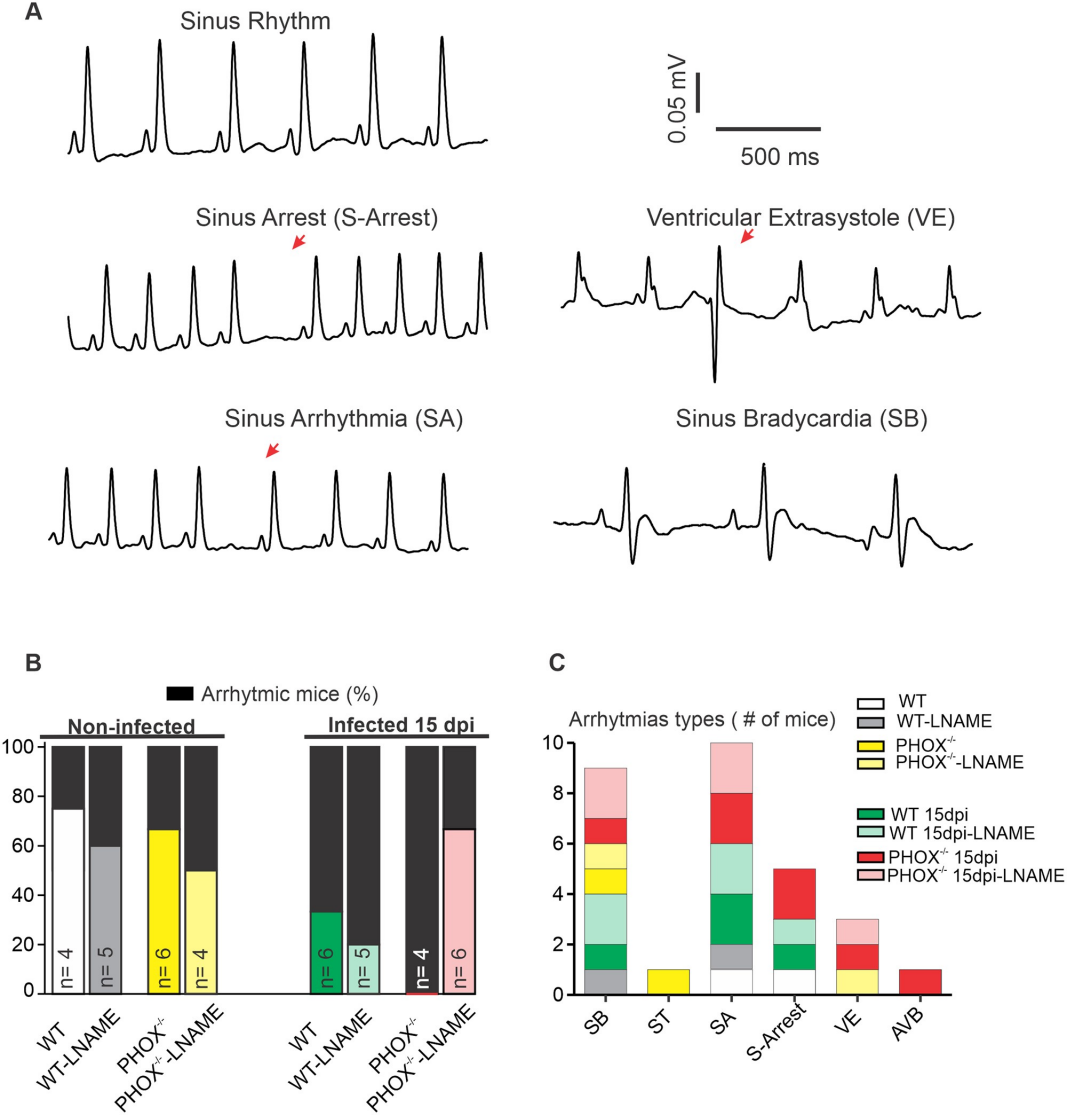
Infected PHOX^-/-^ mice display higher frequency of arrhythmias in the ECG. (A) Representative traces of a sinus rhythm (zoomed central panel) and some types of electrical disturbances recorded, including Sinus stop (upper left, indicated by red arrow); sinus arrhythmia (lower left, red arrow); ventricular extra systole (upper right, red arrow) and sinus bradycardia (lower right). (B) Fraction of mice displaying at least one type of electrical disturbance. Black section indicates presence of any type of arrhythmia. n is the number of mice on each group. (C) Classification of observed arrhythmias among experimental groups, plotted as the number of mice on each group with respect to each given manifestation (Bar size). SB: sinus bradycardia; ST: sinus tachycardia; SA: sinus arrhythmia; S-Arrest: sinus arrest; VE: ventricular extrasystole; AVB: atrioventricular block.

In order to access heart electrical function after acute infection with *T. cruzi*, non-invasive ECG was performed in anesthetized mice. Fig 1A displays representative traces of regular ECG recordings (top panel, zoomed) and some of the arrhythmias observed. As shown in Fig 1B, all experimental groups display some electrical disturbances, and the fraction of mice with electrical disturbances (black bars) in non-infected groups are generally lower than in infected groups. Treatment with L-NAME does not significantly prone the non-infected mice to the appearance of cardiac arrhythmias (p>0.05, Fisher’s exact test). All infected PHOX^-/-^ mice have at least some arrhythmia manifestation, trending towards a significantly more arrhythmogenic profile when compared to non-infected WT or PHOX^-/-^ (p = 0.071). Treatment of these mice during 15 days after infection with a non-specific NOS inhibitor partially prevented the appearance of arrhythmias (to 50% of the mice, p = 0.076). Infected WT mice do not display a significant increase in arrhythmogenic profile when compared to non-infected WT mice (p = 0.261). NOS inhibition did not alter the fraction of arrhythmic mice in infected WT mice (p = 0.575). Finally, despite the ~40% difference in the fraction of arrhythmic mice, infected WT were not significantly different from infected PHOX^-/-^ (p = 0.33). Fig 1C exhibits arrhythmia index classification from all experimental groups as the number of mice with a given manifestation.

### WT and PHOX^-/-^mice display low parasitism with similar tissue remodeling and parasitemia after acute infection with *T*. *cruzi* Y strain

**Fig 2:**
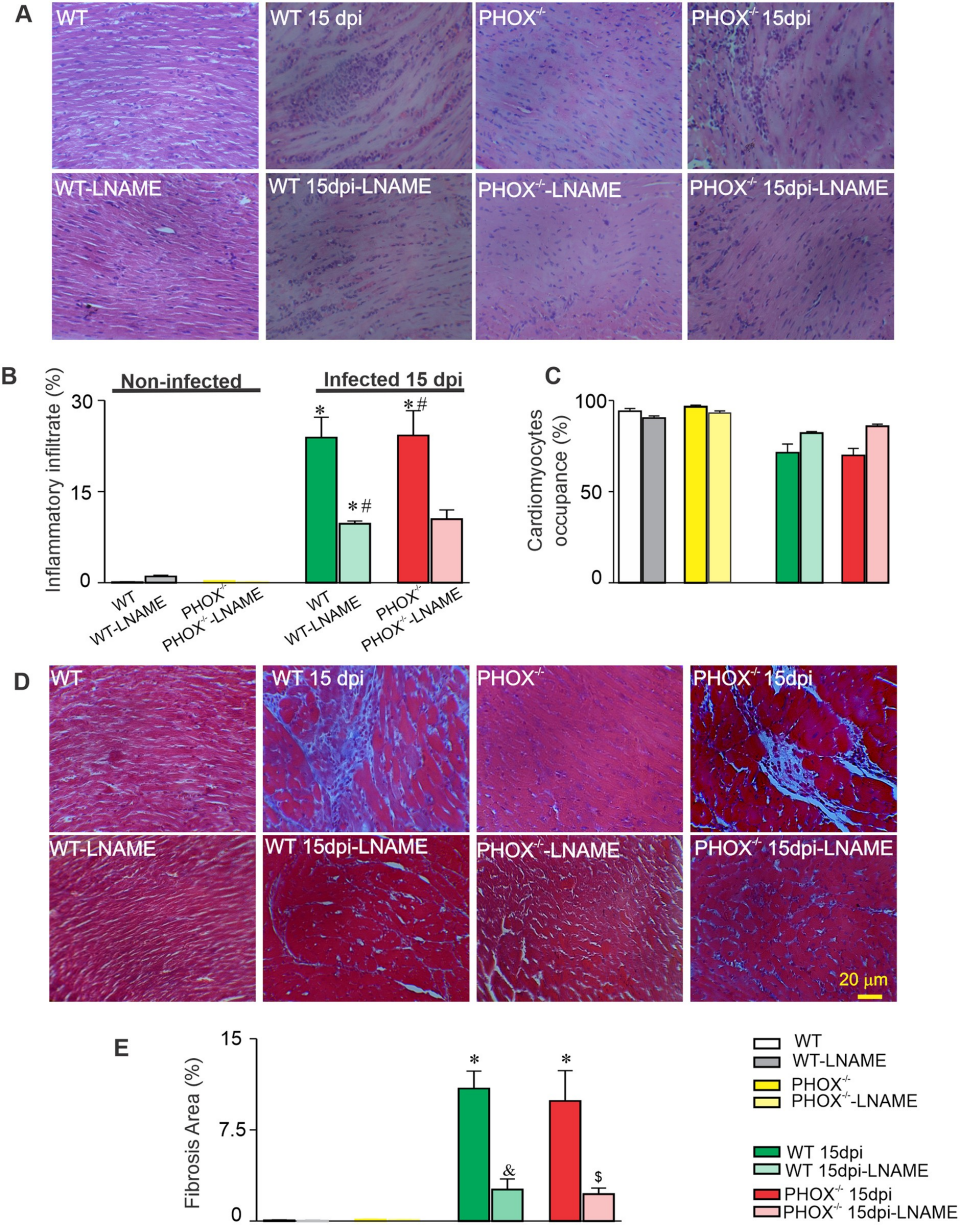
Acute *T*. *cruzi* infection induces extracellular matrix remodeling with larger inflammatory infiltrate in WT and PHOX^-/-^ mice that is partially prevented by NOS inhibition. (A) and (D) are representative images from experimental groups, stained with Eematoxylin-eosin (A) or Masson’ trichrome (D). No parasite nests were observed in infected groups. Summary of morphometric analysis are displayed, highlighting the extension of inflammatory infiltrate (B), cardiomyocytes area occupancy (C) and collagen deposition (E). WT (N = 4); WT+L-NAME (N = 4); WT 15 dpi (N = 4); WT 15 dpi+L-NAME (N = 4)); PHOX^-/-^ (N = 4); PHOX^-/-^+L-NAME (N = 4); PHOX^-/-^ 15 dpi (N = 4) and PHOX^-/-^ 15 dpi+L-NAME (N = 4). *p<0.05, compared to WT; #p<0.05, compared to PHOX^-/-^; &p<0.05, compared to WT 15 dpi. $ p<0.05, compared to PHOX^-/-^ 15 dpi. Data were compared using One way ANOVA’ test followed by Tukey’s posttest. dpi: days post infection. N represents the number of animals.

The increase in arrhythmic events led us to investigate the heart tissue remodeling during the acute phase of experimental infection with *T*. *cruzi*, since electrical disturbances could be linked to extracellular matrix remodeling or discrepant tissue parasitism and inflammation. Histological images were used in order to quantify the parasitism in heart tissue, the amount of inflammatory infiltrate, cardiomyocytes occupancy, and fibrosis area in each section. Fig 2A and 2D are representative images stained with Eematoxylin-eosin and Masson’s trichrome, respectively. No evident parasite nests could be observed in our experimental infected model, indicating low heart tissue parasitism, in accordance with previous results from our group [24]. However, infection with *T*. *cruzi* induced an increase in the inflammatory cell infiltrate in a similar magnitude as for infected WT and PHOX^-/-^ mice (Fig 2B), which led to a reduced cardiomyocyte occupancy (Fig 2C). In addition, collagen deposition was found increased in infected WT and PHOX^-/-^ groups (Fig 2E). Mice treatment with L-NAME prevented extracellular matrix remodeling in both infected groups, with reduced inflammatory infiltrate (Fig 2B) and collagen deposition (Fig 2E).

### Acute *T*. *cruzi* infection elicits impaired AP depolarization upstroke and delays repolarization phase

**Fig 3:**
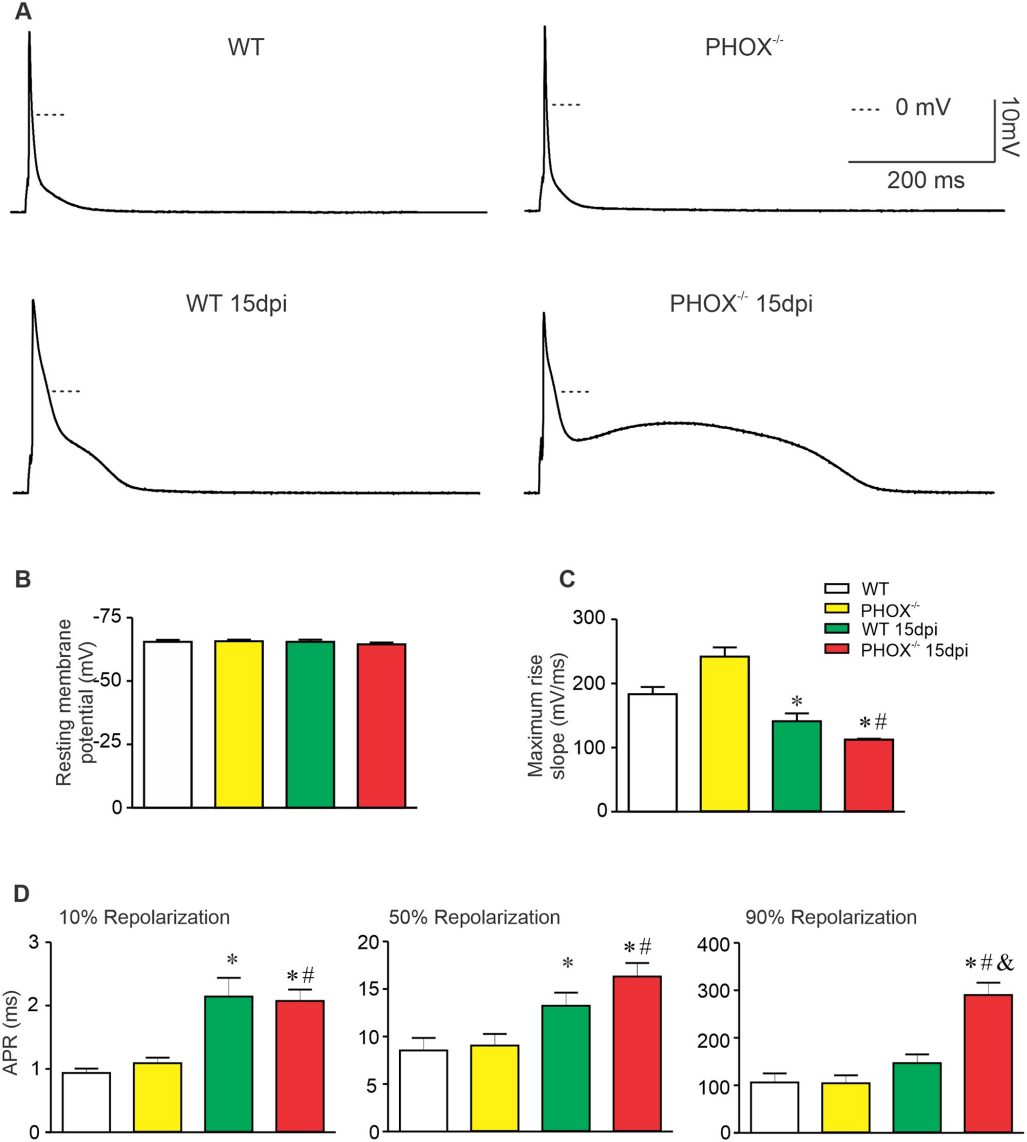
Lack of NOX2-derived ROS in PHOX^-/-^ mice implicates in slower progression on action potential (AP) repolarization in acute phase of chagasic cardiomyopathy. (A) Representative AP recorded from experimental groups, WT (n = 23); WT 15 days post infection (dpi) (n = 32); PHOX^-/-^ (n = 20) and PHOX^-/-^15 dpi (n = 37). (B) Resting membrane potential (given in mV). AP maximum depolarization rates (C). Time required to reach 10%, 50% and 90% of full AP repolarization (D). *p<0.05, compared to WT; #p<0.05, compared to PHOX^-/-^. Data were compared using Kruskal-Wallis’ test followed by Dunns’s posttest. APR: Action potential repolarization; dpi: days post infection. n represents the number of cardiomyocytes.

ROS has long been associated with host attempt to control parasite burn in CD [30,31]. However, increased ROS production is associated with functional and structural damage to heart tissue [12,13,14,18,32] and cardiac outcome as shown to be ameliorated after use of antioxidants [18]. In addition, our group had previously shown that acute experimental CD modulates several ionic conductance in cardiomyocytes which in turn delays AP repolarization and slows depolarization rate [24,33]. Since ROS are important modulators of ion channels, we decided to investigate AP properties in WT and PHOX^-/-^ mice. Left ventricle cardiomyocytes were isolated and patch-clamped in the absence of Ca^2+^ chelators into the patch pipette to study AP properties. During these experiments, a short square pulse (3–5 ms, 1 nA amplitude) was applied to cardiomyocytes to trigger AP at 1 Hz frequency, and membrane voltage waveform was recorded after each pulse. Fig 3A displays representative AP recordings from WT and PHOX^-/-^ mice, non-infected or at 15 dpi. We observed that the resting membrane potential of all experimental groups remains unchanged (Fig 3B). On the other hand, the maximum depolarization rate (dV/dt)_max_ was diminished due to *T*. *cruzi* infection in WT and PHOX^-/-^ groups (Fig 3C). Infected WT and PHOX^-/-^ (15 dpi) have slower AP repolarization compared to non-infected WT and PHOX^-/-^ at 10 and 50% of AP full repolarization (Fig 3D, left and middle panel). However, at 90% AP repolarization (Fig 3D, right panel), only infected PHOX^-/-^ mice display a prolonged AP compared to all other groups. These data indicates that absence of ROS generated from NOX2 lengthened AP repolarization during the acute phase of experimental CD.

### Absence NOX2-derived ROS is associated with AP duration dispersion and increased frequency of EAD

**Fig 4:**
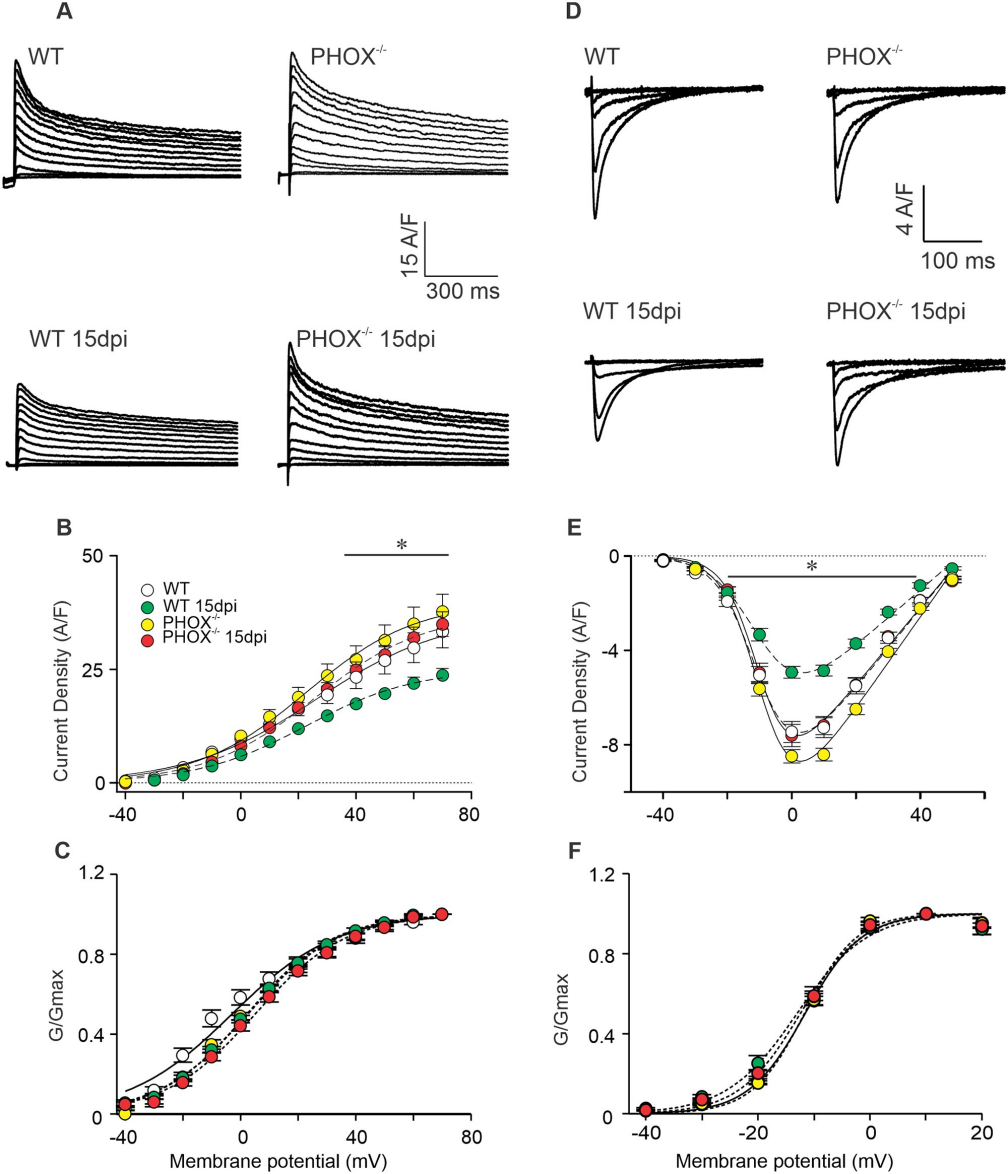
Increased action potential (AP) repolarization dispersion and EAD events in PHOX^-/-^ mice during acute chagasic cardiomyopathy. (A) Four consecutive recorded APs from experimental groups, WT (n = 23); WT 15 days post infection (dpi) (n = 32); PHOX^-/-^ (n = 20) and PHOX^-/-^ 15 dpi (n = 37). EADs are indicated by red arrows. Thirty consecutive APs were analyzed, and the standard deviation (σ) for the time required to reach 90% of AP repolarization was averaged (B) as a measure of AP duration dispersion. (C) Fraction of cells displaying EADs. *p<0.05, compared to WT; #p<0.05, compared to PHOX^-/-^; &p<0.05, compared to WT 15 dpi. Data were compared using Kruskal-Wallis’ test followed by Dunns’s posttest (B) or Chi-squared test (C); σ: Standard deviation; EAD: Early afterdepolarization; dpi: days post infection. n represents the number of cardiomyocytes.

During CD the most problematic clinical complications are the appearance of severe arrhythmias. Here we observed prolongation of AP repolarization, which increases effective cell refractoriness as well as predisposes cardiomyocytes to develop early afterdepolarizations (EADs). In order to further investigate cardiomyocyte propensity to the appearance of arrhythmias, we recorded 30 consecutive AP from each cardiomyocyte and analyzed the appearance of EADs, as well as averaged the time required to reach 90% of full membrane repolarization. Fig 4A displays representative recordings of four consecutive AP recorded from all four experimental groups. The standard deviation (σ) from the time required to reach 90% repolarization of each single AP out of the 30 analyzed was calculated and averaged for each recorded cardiomyocyte (Fig 4B), as a measure of the variation of AP duration. We observed that the standard deviation for 90% repolarization and therefore the dispersion in repolarization time for the same cell is higher for infected PHOX^-/-^ cardiomyocytes compared to all other groups (Fig 4B). Furthermore, the appearance of EADs (Fig 4A red arrows and Fig 4C) is more frequent on AP recorded from infected PHOX^-/-^ compared to all other groups. These data indicate that absence of ROS production from NOX2 increases the likelihood of EADs and AP duration alternans in acute experimental CD, which *per se* augments the susceptibility to develop arrhythmia.

### Absence NOX2-derived ROS prevents acute experimental *T*. *cruzi* infection-dependent reduction of I_Ca-L_ and I_K_ current densities

**Fig 5:**
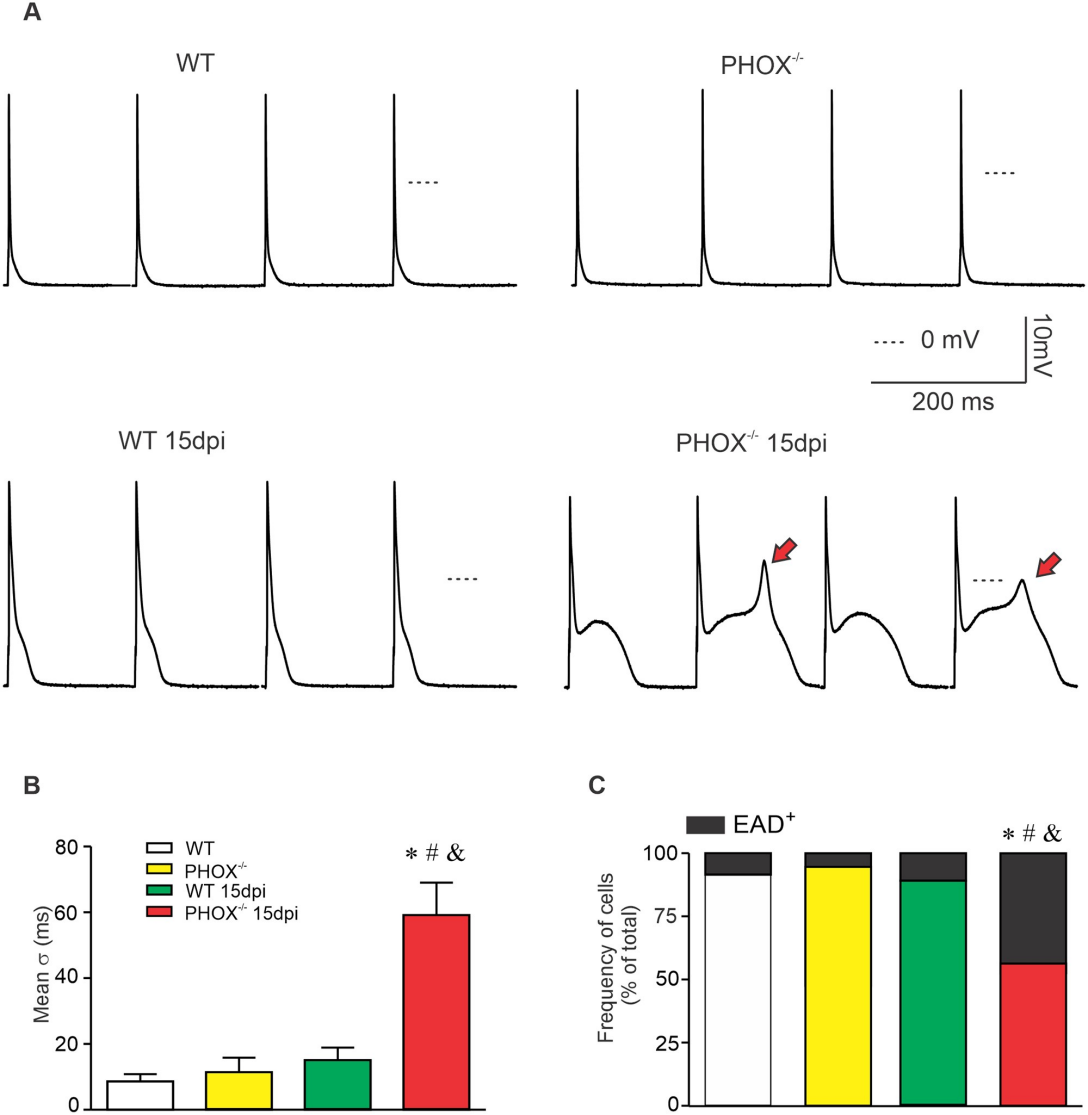
I_Ca-L_ and I_K_ reduction in peak current density during acute phase of chagasic cardiomyopathy is prevented in PHOX^-/-^ mice (A) Representative I_K_ WT (n = 23); WT 15 days post infection (dpi) (n = 23); PHOX^-/-^ (n = 14) and PHOX^-/-^ 15 dpi (n = 16) and I_Ca-L_ (D) traces WT (n = 25); WT 15 days post infection (dpi) (n = 25); PHOX^-/-^ (n = 26) and PHOX^-/-^ 15 dpi (n = 19) recorded from experimental groups. Peak current density from I_K_ (B) and I_Ca-L_ (E) were averaged and plotted against membrane potential. Maximum conductance (G_max_) calculated from current-voltage relationship used to normalize the conductance (G) calculated from each tested potential (C and F). No difference in the voltage dependence for channel activation was observed for I_K_ (C) and I_Ca-L_ (F). *p<0.05, compared to WT. Data were compared using One way ANOVA’ test followed by Tukey’s posttest dpi: days post infection. n represents the number of cardiomyocytes.

We have demonstrated before that in acute experimental CD cardiomyocytes have reduced I_K_ as well as I_Ca-L_ currents. Since voltage-gated Ca^2+^ and K^+^ channels can be modulated by ROS [34,35,36], we decided to investigate how these components are modulated in WT and PHOX^-/-^ cardiomyocytes and how they would contribute to the observed changes in AP waveform. I_K_ currents were elicited through voltage square pulses ranging from -40 to +70 mV (10 mV increments), lasting 3 s, with an interval of 15 s between pulses and starting from a holding potential of -80 mV. I_Ca-L_ currents were studied through a similar stimulation protocol, ranging from -40 to +50 mV, with 3 s duration, and starting from a holding potential of -80 mV. Peak currents were normalized by cell capacitance and plotted as current density. Current density plots were fitted using a Boltzmann-type equation (refer to Methods). Fig 5 depicts representative recordings of total I_K_ (Fig 5A) and I_Ca-L_ (Fig 5B) recorded from WT and PHOX^-/-^ mice, non-infected or at 15 dpi. I_K_ recordings were zoomed in to better emphasize that peak currents have no contribution of capacitive transients or other artifacts during the analyses. We observed a reduction in infected WT mice I_K_ and I_Ca-L_ peak current densities, as it is typical for CD [18,37]. However, the reduction in both current components are prevented in infected PHOX^-/-^ cardiomyocytes, which have similar I_K_ and I_Ca-L_ peak current densities compared to the non-infected groups (Fig 5B and 5E, respectively). Nevertheless, the voltage dependence for I_K_ (Fig 5C) and I_Ca-L_ (Fig 5F) steady-state activation appears to be unchanged during the acute phase of *T*. *cruzi* infection, as evidenced by V_0.5_ and slope (*S*) parameters (Table 1). NOX2-derived ROS play an important role in the reduction of I_Ca-L_ and I_K_ observed in acute CD. Further, it also suggests that AP repolarization lengthening is not influenced by I_K_ or I_Ca-L_, and that other cellular mechanisms are involved in the modulation of AP waveform in infected PHOX^-/-^ mice.

**Table 1:** I_Ca,L_ and I_K_ Boltzmann parameters.

	I_Ca,L_ V_0.5_ (mV)	I_Ca,L_ s		I_K_ V_0.5_ (mV)	I_K_ s
WT	-9.18 ± 0.53	5.65 ± 0.46	WT	1.29 ± 2.25	16.15 ± 2.06
WT 15dpi	-9.86 ± 0.94	6.64 ± 0.77	WT 15dpi	1.82 ± 1.34	15.01 ± 1.22
PHOX^-/-^	-9.84 ± 0.79	5.05 ± 0.71	PHOX^-/-^	1.83 ± 2.21	14.72 ± 1.99
PHOX^-/-^ 15dpi	-9.96 ± 0.96	5.95 ± 0.81	PHOX^-/-^ 15dpi	5.24 ± 1.53	15.07 ± 1.37

*V*_*0*.*5*_: Membrane potential (in mV) at which 50% of the channels are activated. *s*: Slope factor. Data was analyzed using ONE WAY ANOVA with Tukey’s post hoc test.

### Absence of NOX2-derived ROS aggravates Ca^2+^ dysfunction caused by acute *T*. *cruzi* infection in mice

**Fig 6:**
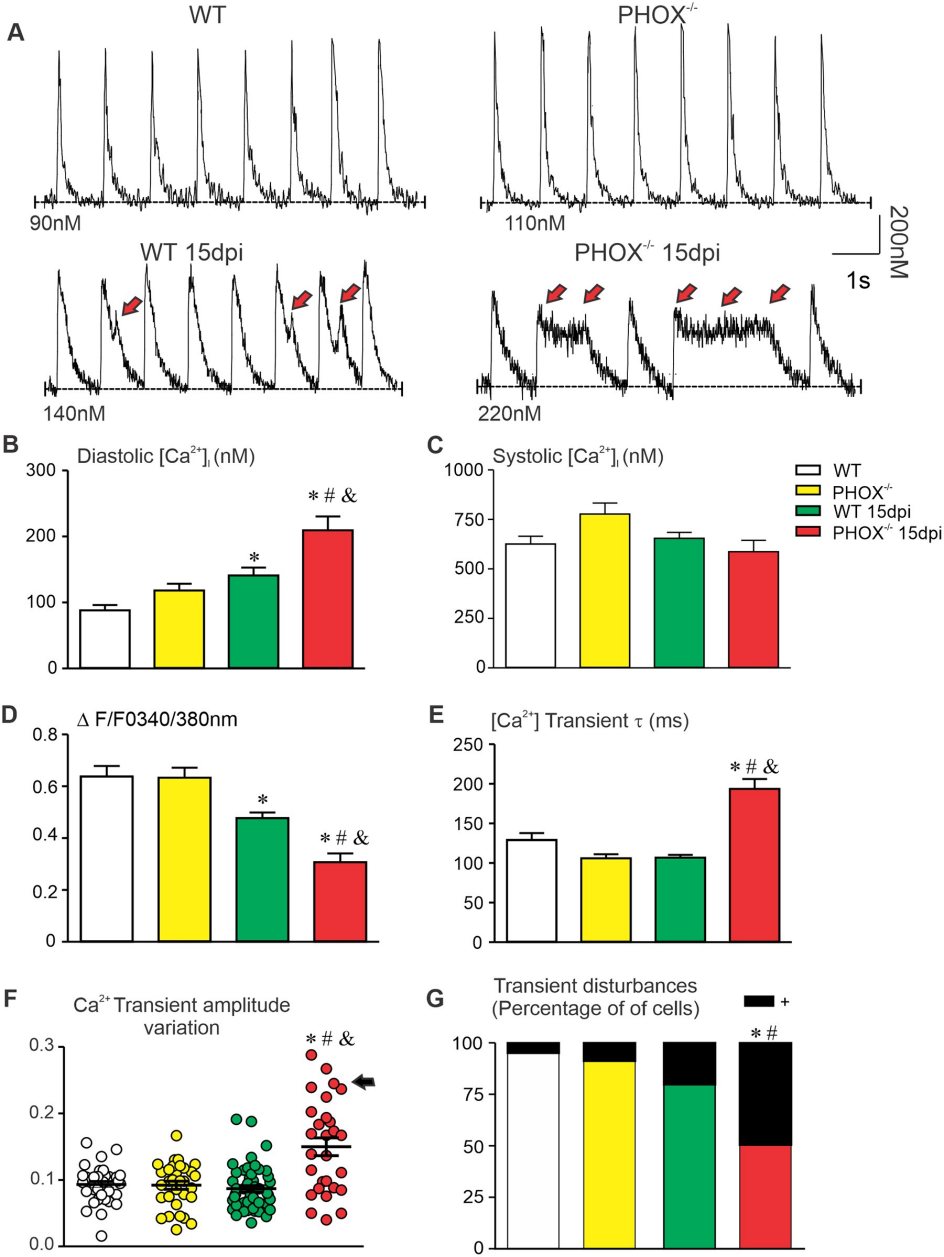
Lack of NOX2-derived ROS in PHOX^-/-^ mice implicates tight modulation of Ca^2+^ dynamics in acute phase of chagasic cardiomyopathy. (A) Eight representative Ca^2+^ transients recorded from experimental groups, WT (n = 36); WT 15 days post infection (dpi) (n = 51); PHOX^-/-^ (n = 31) and PHOX^-/-^ 15 dpi (n = 28). Red arrows indicate disturbances in Ca^2+^ transient. (B) Diastolic and (C) systolic Ca^2+^ concentration. (D) Global Ca^2+^ transient amplitude (ΔF/F_0_). (E) Time constant (τ) of Ca^2+^ transient decay. (F) Thirty consecutive transients were analyzed, and the standard deviation (σ) for peak transient amplitude was averaged. Standard deviation of single cells are plotted individually to highlight the high transient amplitude variation in some cells (black arrow). (G) Fraction of cells with disturbances in the Ca^2+^ transient. *p<0.05, compared to WT; #p<0.05, compared to PHOX^-/-^; &p<0.05, compared to WT 15 dpi. Data were compared using Kruskal-Wallis’ test followed by Dunns’s posttest (B-F) or fisher’s exact test (G) dpi: days post infection. n represents the number of cardiomyocytes.

Ca^2+^ handling is a crucial component to proper control muscle cell function, playing a fundamental role in excitation-contraction coupling. Further, by influencing cardiomyocyte excitation properties it might contribute to the appearance of EADs and increase AP duration alternans [38,39,40,41]. We used the dual excitation Ca^2+^ probe Fura2-AM to investigate Ca^2+^ dynamics on field-stimulated cardiomyocytes. Fig 6A displays global Ca^2+^ transients recorded from WT and PHOX^-/-^ mice, non-infected or at 15 dpi. *T*. *cruzi* infection promoted a significant increase in diastolic Ca^2+^ concentration in both infected groups compared to its respective controls, as demonstrated in Fig 6B. However, diastolic Ca^2+^ concentration on infected PHOX^-/-^ cardiomyocytes is significantly higher even when compared to infected WT mice. On the other hand, when cells are electrically stimulated, the peak Ca^2+^ concentration does not change in WT and PHOX^-/-^mice, even at 15 dpi (Fig 6C). Such Ca^2+^ dynamic profile determines a reduced relationship between peak (F) and diastolic Ca^2+^ concentration (F_0_) in infected groups, WT and PHOX^-/-^mice. F and F_0_ obtained from fluorescence measurements F_340nm_/F_380nm_. Infected PHOX^-/-^F/F_0_ is actually more reduced when compared to infected WT mice (Fig 6D). Furthermore, infected PHOX^-/-^ mice have a slower Ca^2+^ transient decay compared to all other groups, which did not differ from each other, as evidenced by the comparison of calculated time constant for Ca^2+^ transient decay (Fig 6E). Finally, 30 consecutive Ca^2+^ transient amplitudes were measured and their variation analyzed. PHOX^-/-^ cardiomyocytes showed increased variation in Ca^2+^ transient amplitude, with some cells (20%) displaying a very high degree of variation (more them 200% higher) (Fig 6F, black arrow and shown in Fig 6A, right column, lower panel), which does not occur in their respective control group. The fraction of cells displaying changes in Ca^2+^ transient decay was also analyzed (Fig 6G and red arrows on the representative recordings of WT 15 dpi and PHOX^-/-^15 dpi, Fig 6A). PHOX^-/-^cardiomyocytes presented a larger fraction of cells displaying Ca^2+^ transient decay disturbances, compared to non-infected groups. Although approximately 20% of WT 15 dpi had also displayed disturbances in Ca^2+^ transient decay, it was not significantly different from other groups. These data demonstrate that absence of NOX2-derived ROS promotes a cellular environment suitable to the appearance of EADs and AP duration alternans that were observed in infected PHOX^-/-^ cardiomyocytes.

### Cardiomyocyte sarcomere contraction is impaired in the acute phase of experimental CD, with further increase of extra-contractions in the absence of NOX2-derived ROS

**Fig 7:**
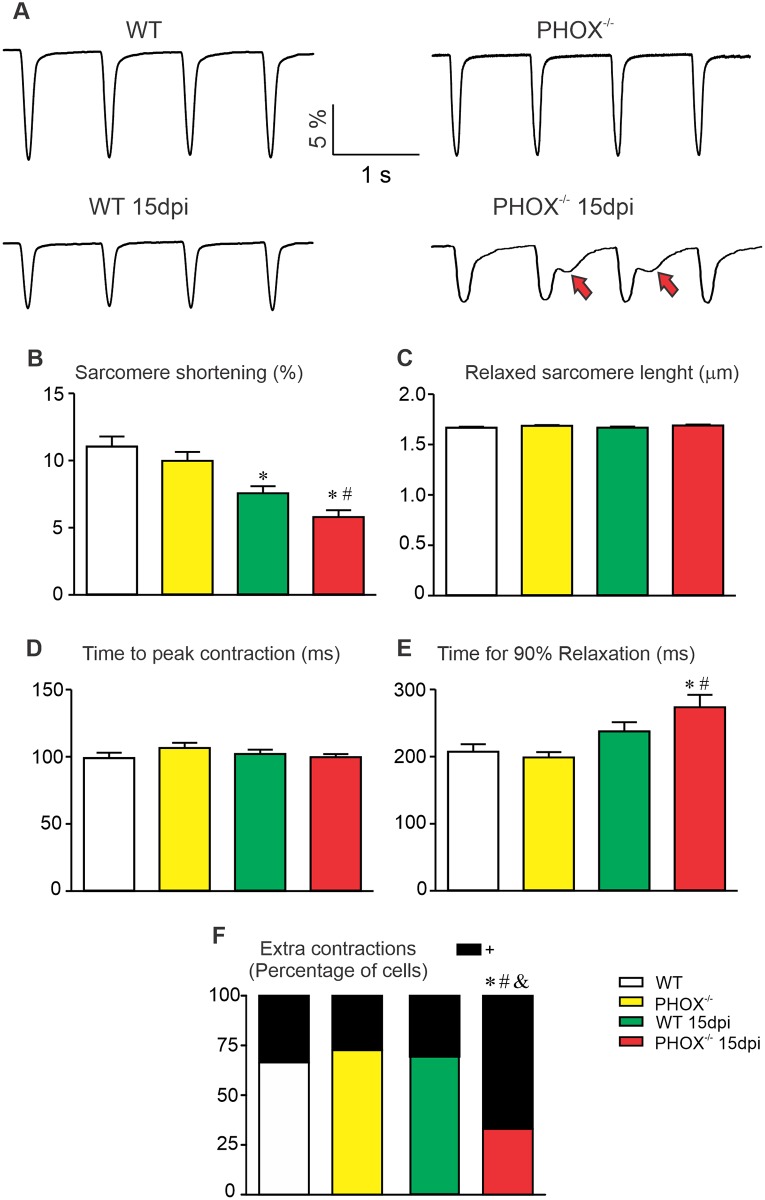
Impairment of cell contraction during acute phase of chagasic cardiomyopathy. (A) Four representative sarcomere contraction recorded from experimental groups WT (n = 32); WT 15 days post infection (dpi) (n = 49); PHOX^-/-^ (n = 29) and PHOX^-/-^ 15 dpi (n = 30). Red arrows indicate extra-contractions that appear without external stimulation. (B) Sarcomere contraction percentage. (C) Sarcomere length in the relaxed state. (D) Time required to reach maximum contraction from full relaxed state. (E) Time required to recover 90% of full relaxed state. (F) Fraction of cells in which extra-contractions was observed. *p<0.05, compared to WT; #p<0.05, compared to PHOX^-/-^; &p<0.05, compared to WT 15 dpi. Data were compared using Kruskal-Wallis’ test followed by Dunns’s posttest (B-E) or fisher’s exact test (F) dpi: days post infection. n represents the number of cardiomyocytes.

In order to further investigate cardiomyocyte function, sarcomere shortening and re-lengthening were evaluated on field-stimulated cardiomyocytes through sarcomere deconvolution-based analyses using a fast Fourier transform. Sarcomere size and the appearance of extra-contractions, described as contractions apart from the field-stimulated ones were monitored using a sarcomere detection system (see Methods). We observed a reduction in the fractional shortening of sarcomeres from infected WT and PHOX^-/-^ cardiomyocytes, when compared to their respective non-infected controls, as demonstrated in the representative recordings (Fig 7A) and in the averaged plot (Fig 7B). Fractional shortening was considered as the ratio between peak contraction and fully relaxed sarcomere. Sarcomere size was not modified in all studied groups (Fig 7C). In addition, the time required to reach peak contraction was similar between all groups (Fig 7D), while sarcomere relaxation, measured as the time required to reach 90% relaxation from peak contraction, was found to be increased in infected PHOX^-/-^cardiomyocytes compared to all other groups (Fig 7E). Cell dimensions do not differ among experimental groups: WT (125.8 ± 2.4 μm, n = 49), infected WT (126.1 ± 4.8 μm, n = 33), PHOX^-/-^ (115.7 ± 2.3 μm, n = 45) and infected PHOX^-/-^ (113.3 ± 3.7 μm, n = 30). Finally, extra-contractions appeared more frequently in infected PHOX^-/-^ cardiomyocytes (Fig 7F).

### Inhibition of NO production prevents the AP alternans and increased EAD events on infected PHOX^-/-^ cardiomyocytes

**Fig 8:**
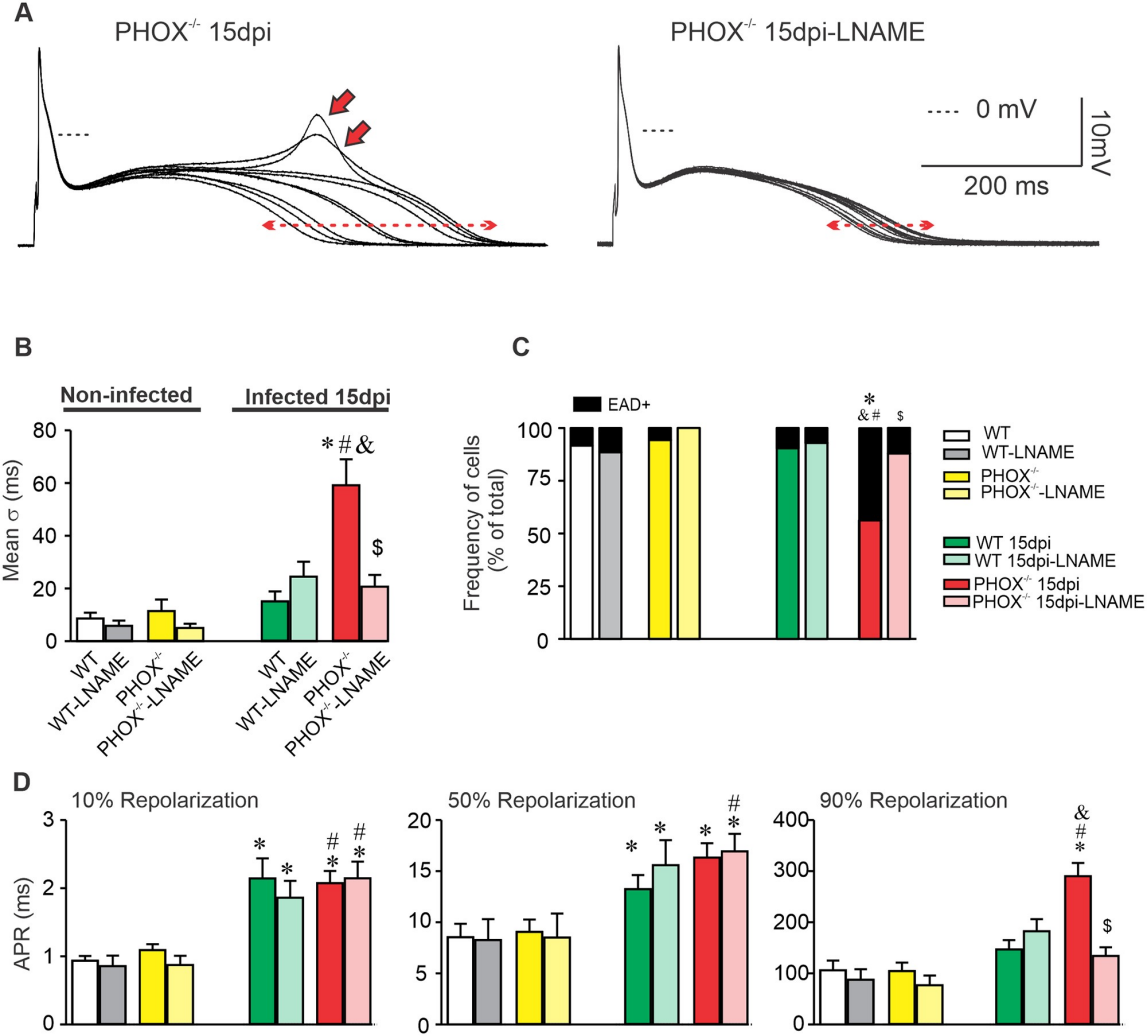
Inhibition of NO production prevents the appearance of EADs and the increase in action potential (AP) duration dispersion observed in PHOX^-/-^ mice during acute phase of chagasic cardiomyopathy. (A) Ten superimposed AP recorded from infected PHOX^-/-^ cardiomyocytes in absence (left panel) or after inhibition of NO production with L-NAME (right panel). Red arrows indicate the appearance of EADs, while dashed red lines with arrowheads indicate magnitude of AP duration dispersion. (B) AP duration dispersion and (C) Fraction of cells displaying EADs with and without inhibition of NO production. (D) Time to reach 10% (left panel), 50% (middle panel) and 90% (right panel) of full membrane repolarization in response to NO inhibition. WT (n = 23); WT+L-NAME (n = 9); WT 15 days post infection (dpi) (n = 32); WT 15 dpi+L-NAME (n = 14); PHOX^-/-^ (n = 20); PHOX^-/-^+L-NAME (n = 10); PHOX^-/-^ 15 dpi (n = 37) and PHOX^-/-^ 15 dpi+L-NAME (n = 17). *p<0.05, compared to WT; #p<0.05, compared to PHOX^-/-^; &p<0.05, compared to WT 15 dpi. $ p<0.05, compared to PHOX^-/-^ 15 dpi. Data were compared using Kruskal-Wallis’ test followed by Dunns’s posttest. APR: Action Potential Repolarization; σ: Standard deviation; and EAD: Early afterdepolarization; dpi: days post infection. n represents the number of cardiomyocytes.

Our ECG analyses suggest that cardiomyocyte dysfunction could be due to higher NO production in the absence of superoxide production in PHOX^-/-^mice. In order to test this hypothesis, AP was recorded on a set of cells to which 10 μM L-NAME was added to the Tyrode solution for 30 minutes prior to manipulation, in order to inhibit the production of NO [24]. Fig 8A represents an example of ten superimposed consecutive AP recorded from an infected PHOX^-/-^ cardiomyocyte, in the absence (left panel) or after 30 minutes incubation with L-NAME (right panel). Inhibition of NO production prevented the appearance of EADs as well as reduced dispersion in the AP alternans (Fig 8B and 8C). Moreover, we observed a slower progression of AP repolarization in infected WT and PHOX^-/-^ mice at 10% and 50% after incubation with L-NAME (Fig 8D). However, L-NAME significantly reduced the time required to reach 90% of AP repolarization in infected PHOX^-/-^ mice. These data indicate that cardiomyocyte dysfunction found in infected PHOX^-/-^ mice may be related to higher NO production.

## Discussion

In this study, we used a mouse model (PHOX^-/-^) lacking functional NOX2 to assess the effects of NOX2-derived ROS on the outcome of cardiac electrical and mechanical functions during the acute phase of CD. The lack of NOX2-derived ROS is validated by the absence of increase in macrophage ROS production when stimulated with either *T*. *cruzi* or zymosan (S2 Fig). Absence of NOX2-derived ROS creates an arrhythmogenic environment that is neither associated with increased tissue parasitism nor extracellular matrix remodeling compared to infected WT mice. However, PHOX^-/-^ isolated cardiomyocytes displayed profound remodeling of AP and Ca^2+^ handling properties after *T*. *cruzi* infection. The increased frequency of EADs and AP duration alternans, followed by increased occurrence of global Ca^2+^ transient disturbances and peak alternans lead to a pro-arrhythmogenic profile in infected PHOX^-/-^ mice. The pro-arrhythmogenic substrate observed was supported by an increase in extra-contractions from infected PHOX^-/-^ cardiomyocytes. Interestingly, the arrhythmogenic profile of PHOX^-/-^ was reverted by NOS inhibition in both *in vivo* and *in vitro* experiments.

Among the most common and severe clinical manifestations of CD are the presence of cardiac arrhythmias [4,7,42,43]. Although severe and diverse arrhythmic manifestations are often seen in chronic phase of CD [4,7] approximately 5% of chagasic patients die during acute *T*. *cruzi* infection due to cardiac failure, encephalomyelitis and sudden death [7]. Our work focuses on the acute phase of experimental CD, which does not fully recapitulate the reactivation of the chronic phase of the disease, when structural remodeling of the heart is more prominent [7]. Nevertheless, oral transmission of CD through ingestion of contaminated food is associated with more complicated clinical forms and higher mortality [9], with severe cases reported from both in acute [44] and chronic [45] phases.

Sudden death has been linked to the appearance of cardiac arrhythmias [46], which could account for the mortality in the acute phase of CD. In our study we observed spontaneous arrhythmic events in all experimental groups. However, infected mice have an increased frequency of electrical disturbances and a broader range of arrhythmic manifestations, including ventricular extra-systoles and atrio-ventricular block (AVB) that are typical forms of arrhythmia seen in chagasic patients [7]. Importantly, all infected PHOX^-/-^ mice had at least one arrhythmic manifestation, and this arrhythmic profile was partially prevented by NOS inhibition after *T. cruzi* infection. On the other hand, NOS inhibition could not attenuate the arrhythmogenic profile of infected WT mice, suggesting that simply reducing NO levels does not provide an antiarrhythmic scenario during the acute phase of experimental CD. Spontaneous electrical disturbances observed in non-infected groups could be linked to the anesthetics used in the experiments, especially considering that xylazine [47] and ketamine [48] have been shown to modulate several ion channels. In addition, xylazine-ketamine combination can trigger heart electrical disturbances during ECG recordings, particularly bradycardia [49].

ECG electrical disturbances are associated with modulation of cardiomyocytes excitability, structural remodeling of the heart tissue, or a combination of both factors [50]. Structural remodeling during CD is often linked to loss of cardiomyocytes and extracellular matrix remodeling with collagen deposition and inflammatory infiltrate [2]. All these factors lead to electrical conduction problems therefore creating an arrhythmogenic substrate in the heart. We did not observe differences in inflammatory infiltrate, cardiomyocyte occupancy, or collagen deposition between infected WT and PHOX^-/-^ mice, suggesting that loss of cardiomyocytes or extracellular matrix remodeling is not the main mechanism driving the electrical dysfunction observed in PHOX^-/-^ mice. Tissue parasitism was also low in all infected groups, and no parasite nests were observed in infected groups, excluding the hypothesis that increased parasitism could lead to the appearance of arrhythmic hearts in PHOX^-/-^ mice. In line with this rationale, our group previously demonstrated that PHOX^-/-^ mice have similar tissue parasitism and immune response compared to infected WT mice during the acute phase [24]. Here we also show that parasitemia is comparable between these infected groups (S2 Fig). NOS inhibition prevented extracellular matrix remodeling and attenuated the inflammatory infiltrate in both infected groups. However, NOS inhibition did not attenuate the fraction of infected WT mice presenting arrhythmias. This finding reinforces the idea that extracellular matrix remodeling is not the key factor triggering arrhythmias in our model of acute CD. It is important to highlight that during chronic CD extracellular matrix remodeling provokes significant structural changes [5,7,42], that are followed by the appearance of electrical disturbances, including reentry arrhythmias and conduction blockade [5,7,42].

Regarding modulation of cardiomyocyte excitability, our group and others have consistently demonstrated AP waveform remodeling during acute and chronic phases of experimental CD [18,37,51]. Interestingly, this AP remodeling was also reported when cardiomyocytes were exposed to auto-antibodies from chagasic patients [52]. Among the most common findings are prolongation of the AP repolarization most probably due to a reduction in outward potassium currents (I_K_) [18,37]. These observations are in accordance with our findings. In addition, reduced L-type Ca^2+^ current density (I_Ca-L_) was also observed in acute experimental infection using Colombian and Y *T*. *cruzi* strains [18,37,51], similarly to what was described in the present study. Surprisingly, infected PHOX^-/-^ mice have comparable I_K_ and I_Ca-L_ when comparing both non-infected and control groups. Transient outward potassium current (I_to_), one of the major I_K_ components in murine ventricular cardiomyocytes [53], is reduced under increased oxidative status [54,55]. PHOX^-/-^ mice lack NOX2 contribution to global ROS generation [23] and NOX2 is one of the most important ROS producers during *T*. *cruzi* infection [25]. It is suggested that lack of NOX2-derived ROS in cardiomyocytes prevented the reduction of the peak I_to_ current density. Similarly, there are consolidated evidences of ROS-dependent reduction of I_Ca-L_ in cardiomyocytes [56,57,58], which could account for the observed reduction in infected WT but not in infected PHOX^-/-^ mice. On the other hand, it is important to state that NO has also been suggested to play a role in reducing both I_to_ [59], and I_Ca-L_ [60]. In the present study, we observed high NO levels in isolated cardiomyocytes of infected PHOX^-/-^ mice compared to all other groups (S3 Fig). Nevertheless, the interplay between molecular mechanisms driving the modulation of I_K_ and I_Ca-L_ and its relationship with cardiomyocyte oxidative status are still controversial and need to be better elucidated.

Despite the fact that we did not observe important differences in I_K_ and I_Ca-L_ peak current densities in infected PHOX^-/-^ cardiomyocytes, we did observe a pronounced delay in AP repolarization, which one would attribute to other factors. In our experimental model of acute CD, we observed an increased diastolic Ca^2+^ concentration of infected PHOX^-/-^ cardiomyocytes compared to all other groups. It is interesting to note that NO [21,53] and ROS [61,62,63] modulate ryanodine receptors (RyR) increasing open probability (P_open_RyR). An increase in sarcoplasmic reticulum (SR) Ca^2+^ leak, in turn, would have contributed to the observed high diastolic Ca^2+^ concentration, and because RyR are activated by sarcoplasmic Ca^2+^, the increase in diastolic Ca^2+^ concentration may also promote an increase in SR Ca^2+^ leak [38,63,64], further aggravating the diastolic dysfunction. Strengthening this hypothesis, SR Ca^2+^ leak may be determinant to the appearance of EADs and delayed afterdepolarizations (DADs), as a consequence of RyR Ca^2+^ leak with subsequently Na^+^/Ca^2+^ exchanger (NCX)-driven membrane depolarization and reactivation of I_Ca-L_ (for a review, see: [38,39,40]). This is especially evident with prolonged AP repolarization phase [39,40]. At this point it is important to note that in addition to Na^+^ and Ca^2+^ electrochemical gradients, NCX also has its activity allosterically activated by Ca^2+^ [38,65]. Because NCX current (I_NCX_) is electrogenic, in such environment of increased RyR P_open_ and higher SR Ca^2+^ leak, an increase in intracellular Ca^2+^ favors its function in forward direction providing a resultant vector towards membrane depolarization and hence AP prolongation, which is totally consistent with our findings. Finally, AP duration alternans [41,66,67], as well as disturbances in Ca^2+^ handling [68,69,70], have been associated with the occurrence of cardiac alternans, which pre-disposes appearance of arrhythmias. This is consistent with the finding that all infected PHOX^-/-^ mice displayed electrical disturbances on the ECG readings.

In our study, we observed a reduction in Ca^2+^ transient decay and a delay in sarcomere relaxation, which reflects slower Ca^2+^ reuptake to SR (and therefore lower Sarco(Endo)plasmic Reticulum Ca^2+^ ATPase (SERCA) activity), together with increased diastolic Ca^2+^ concentration. One would likely assume that infected PHOX^-/-^ cardiomyocytes have an increase in SR Ca^2+^ leak associated with slow Ca^2+^ re-uptake to the SR, triggering the alternans observed in Ca^2+^ transients and AP duration, as well as the appearance of EADs in a prolonged AP that arises probably in response to I_NCX_. In this scenario, the disturbances in SR Ca^2+^ release and re-uptake may also lead to the reduced average global Ca^2+^ transients, which lead to reduced sarcomere contraction that is observed in infected PHOX^-/-^ mice. In agreement with our previous results, the appearance of extra-contractions is correlated with the high frequency of EADs and the overall pro-arrhythmogenic profile of infected PHOX^-/-^.

Cardiomyocytes from infected WT mice, on the other hand, have a slight increase in diastolic Ca^2+^ compared to non-infected WT. However, we did not observe statistical differences in the alternans of Ca^2+^ transient and AP duration. These cardiomyocytes have increased ROS production as indicated by increased DHE fluorescence compared to all other groups (S3 Fig). Because RyR P_open_ is modulated by ROS [61,62,63], it is possible that the ROS-induced increase in its opening probability could lead to an increase in diastolic Ca^2+^ concentration in response to increased SR Ca^2+^ leak [63], as well as an increase in systolic Ca^2+^ release. However, because of the diminished I_Ca-L_, Ca^2+^-induced Ca^2+^ release from SR might mitigate the effects of ROS on RyR P_open_, leading to a comparable systolic Ca^2+^ concentration. Nevertheless, the net result of increased diastolic Ca^2+^ with no changes in Ca^2+^ systolic concentration leads to a reduced Ca^2+^ transient amplitude and sarcomere contraction. The time to peak sarcomere contraction remained the same for all groups, suggesting that if there was an asynchrony of Ca^2+^ release from RyR in this mice model as our group has already demonstrated during acute phase of experimental CD as a result of *T*. *cruzi* Colombian strain [18] infection, it may be not sufficient to provoke a contraction delay.

We found that, during infection, both WT and PHOX^-/-^ mice have increased superoxide production in mitochondria (S3 Fig), in agreement with previous reports on mitochondrial dysfunction during *T*. *cruzi* infection, leading to increased production of ROS [10,11,12,71]. Cytosolic superoxide production is also increased in both infected groups compared to the respective non-infected controls. However, the extent of total superoxide anion production in infected WT is much higher compared to infected PHOX^-/-^ mice, as would be expected from mice lacking functional NOX2. The increase in cytosolic superoxide production observed in PHOX^-/-^ could be associated with other NOS isoforms and other cellular systems, including xanthine oxidase-derived ROS and mitochondrial leak. In fact, mitochondrial ROS increase in infected PHOX^-/-^ was higher when compared to infected WT mice, supporting a larger mitochondrial ROS leak of these species. Superoxide combines with NO to generate peroxynitrite [72], one of the most important effectors to fight *T*. *cruzi* [73]. We have demonstrated an increase in NOS expression isoforms in cardiomyocytes during experimental *T*. *cruzi* infection [18]. In the present study, infected WT mice displayed reduced NO levels compared to their non-infected controls. We speculate that it could be the result of NO sink after peroxynitrite formation, especially in an environment of increased superoxide availability that is found in our infected WT mice. This is the reciprocal rationale from what was suggested in another publication from our group [24]. In line with this rationale, our results indicate that infected PHOX^-/-^ cardiomyocytes have lower amounts of net superoxide compared to infected WT while they have higher levels of NO compared to all other groups.

Most of the cellular machinery involved with cardiomyocyte excitation-contraction coupling (ECC) discussed above are known to be modulated by NO and ROS. Interestingly, inhibition of NO prevented the overall arrhythmogenic environment on infected PHOX^-/-^ mice, both *in vivo* and *in vitro*. These findings demonstrate that unbalanced NO levels worsens the outcome of experimental CD, suggesting that not only the amount of ROS/NO but the stoichiometry between them might be determinant to the outcome of CD prognosis. With that in mind, here we provide evidence that NOX2-derived ROS is important to balance NO overproduction during *T*. *cruzi* infection, and that inhibition of NOX2, alone, worsens the cardiac outcome, even though other studies have demonstrated that controlling ROS production through NOX2 [74] or through NOX in general [32] controls *T*. *cruzi*-induced infection and ameliorates myocarditis. Treatment with non-specific antioxidants were shown to be effective to prevent [17] or to revert [18] experimental CD. Yet, ROS and NO have important signaling roles in tuning cardiomyocytes’ electrical [20,21] and contractile [22] functions that might be off target using general non-specific approaches. Besides, it is already known that increased levels of NO and cytokines (e. g TNF, IL1B and IL6) are positively correlated with severity of experimental and human chagasic cardiomyopathy and inducible isoform of NO is important for the observed arrhythmogenic phenotype. Thus, future studies targeting specific NOS and mitochondrial ROS sources should be conducted in order to provide evidence for a more specific therapeutic approach, controlling parasite proliferation while also ameliorating disease symptoms, especially during the chronic phase of CD.

Taken together, our data provide evidence that the outcome of the cardiac function during acute experimental CD is dependent on the NO and ROS balance. Ablation of NOX2 creates an arrhythmogenic environment that is associated with *in vivo* arrhythmias which is related to cellular electrical remodeling as well as Ca^2+^ handling disturbances in cardiomyocytes. Importantly, neither heart tissue remodeling nor increased parasitism load seems to play a key role. Lastly, such pro-arrhythmogenic substrate was mostly due to excessive NO production by the cardiomyocytes. Therefore, complete scavenging NOX2-derived ROS in the myocardium during the development of CD may not provide a suitable therapeutic target to treat chagasic cardiomyopathy.

## Supporting information

S1 Fig Example of histological morphometric analysis using a pre-defined grid and a cell counter tool for measuring: inflammatory infiltrate (demarcated by number 1); cardiomyocyte nuclei (demarcated by number 2); cardiomyocyte fiber (demarcated by number 3), blood vessels (demarcated by number 4). Cardiomyocyte occupancy was calculated by the sum of cardiomyocyte nuclei total number and cardiomyocyte fiber total number. The final values were expressed in percentage. A total of 1,000 grid intersection points were analyzed in different sections per animal. The ImageJ software was used for the grid construction and analysis.
(TIF)

S2 Fig PHOX^-/-^ mice lacking functional NOX2 have similar parasitemia but higher mortality index compared to WT when infected with *T*. *cruzi* Y strain. Mice were infected with 1000 blood-born trypomastigotes of Y strain of *T*. *cruzi*. Parasitemia (A) and mortality (B) were accessed daily. (A) Points represent mean ± SE of five animals per group from three different experimental infections. (B) Mortality curve is pooled from three experimental infections. C-D: Production of reactive oxygen species by macrophages stimulated with *T*. *cruzi*. Macrophages were incubated with 0.5 mM of luminol in culture medium and exposed to *T*. *cruzi* trypomastigotes or zymosan. Chemiluminescence was measured immediately and every 2 min, for 120 min (C) The area under curves, representing total ROS production over time was calculated (D) and plotted as mean± S.D. Graphs are representative of three independent experiments performed in triplicate (cells were pooled from three mice for each replicate). * refers to significant differences from the infected and zymosan treated to non-treated macrophages. Data were compared using 2-way ANOVA followed by Bonferroni post hoc test (A-B) or one way ANOVA followed by tukey’s post hoc test (C-D) *p<0.05, compared to WT. RFU: Relative fluorescence units.
(TIF)

S3 Fig Lack of NOX2-derived ROS in PHOX^-/-^ mice implicates imbalances in NO and superoxide production during acute phase of chagasic cardiomyopathy. (A) Mitochondrial superoxide production was accessed using 5 μM of MitoSOX probe. WT (n = 94); WT 15 days post infection (dpi) (n = 48); PHOX^-/-^ (n = 100) and PHOX^-/-^ 15 dpi (n = 108). (B) Total production of superoxide, accessed with dihydroethidium probe 5 μM WT, (n = 121); WT 15 dpi, (n = 60); PHOX^-/-^, (n = 107) and PHOX^-/-^ 15 dpi, (n = 66). (C) NO production, accessed with DAF-FM 5 μM: WT, (n = 94); WT 15 dpi (n = 82); PHOX^-/-^ (n = 112); and PHOX^-/-^ 15 dpi, (n = 117). *p<0.05, compared to WT; #p<0.05, compared to PHOX^-/-^; &p<0.05, compared to WT 15 dpi. Data were compared using Kruskal-Wallis’ test followed by Dunns’s posttest and plotted as fluorescence arbitrary units (A.U). n represents the number of cardiomyocytes.
(TIF)
