# Correction: Reactive oxygen species and nitric oxide imbalances lead to *in vivo* and *in vitro* arrhythmogenic phenotype in acute phase of experimental Chagas disease

**DOI:** 10.1371/journal.ppat.1009049

**Published:** 2020-10-28

**Authors:** Artur Santos-Miranda, Julliane Vasconcelos Joviano-Santos, Grazielle Alves Ribeiro, Ana Flávia M. Botelho, Peter Rocha, Leda Quercia Vieira, Jader Santos Cruz, Danilo Roman-Campos

Figs 4 and 5 are switched and are listed under the incorrect legend. Please see the correct figures and legends below.

**Fig 4:**
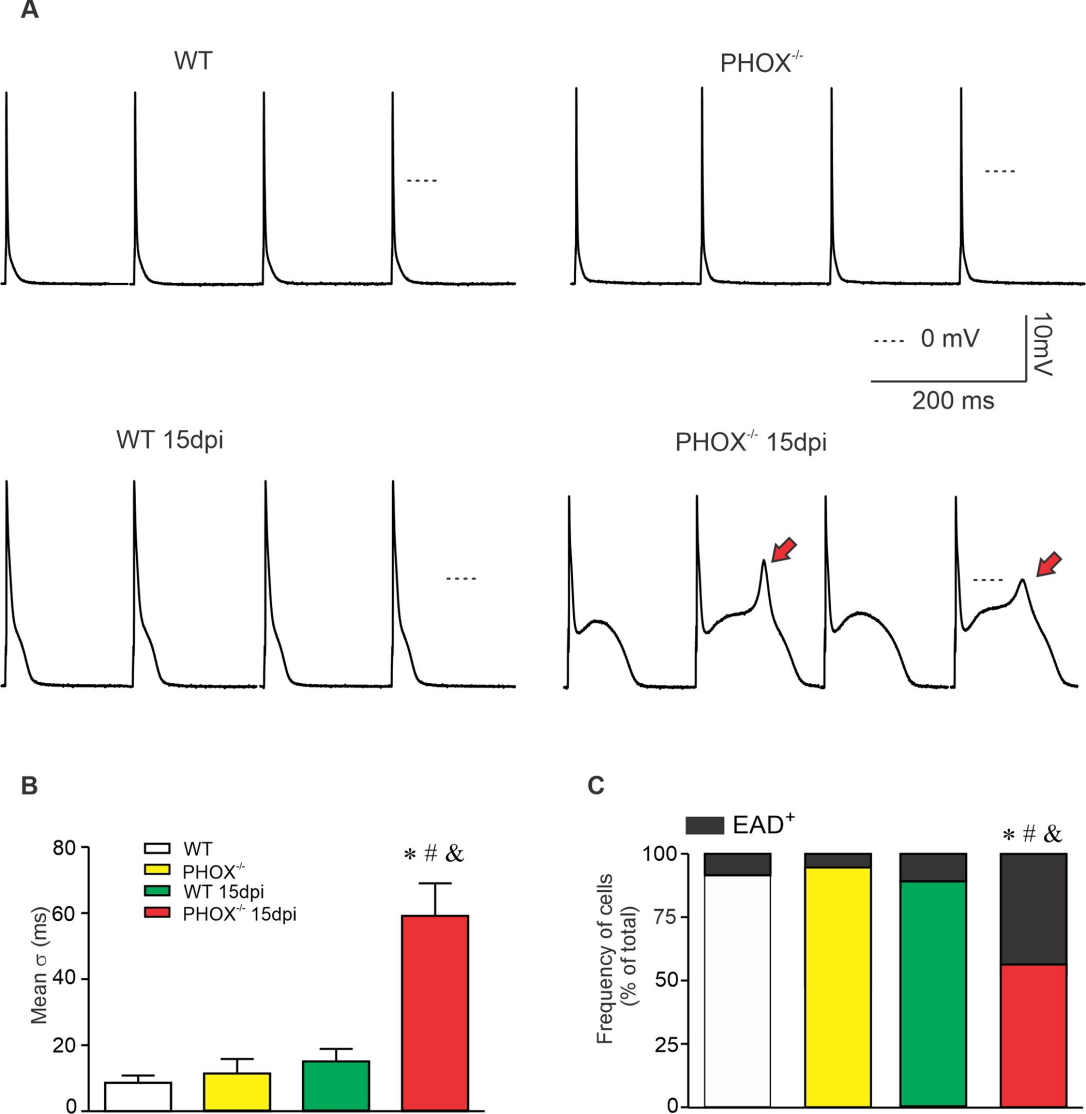
Increased action potential (AP) repolarization dispersion and EAD events in PHOX^-/-^ mice during acute chagasic cardiomyopathy. (A) Four consecutive recorded APs from experimental groups, WT (n = 23); WT 15 days post infection (dpi) (n = 32); PHOX^-/-^ (n = 20) and PHOX^-/-^ 15 dpi (n = 37). EADs are indicated by red arrows. Thirty consecutive APs were analyzed, and the standard deviation (σ) for the time required to reach 90% of AP repolarization was averaged (B) as a measure of AP duration dispersion. (C) Fraction of cells displaying EADs. *p<0.05, compared to WT; #p<0.05, compared to PHOX^-/-^; &p<0.05, compared to WT 15 dpi. Data were compared using Kruskal-Wallis’ test followed by Dunns’s posttest (B) or Chi-squared test (C); σ: Standard deviation; EAD: Early afterdepolarization; dpi: days post infection. n represents the number of cardiomyocytes.

**Fig 5:**
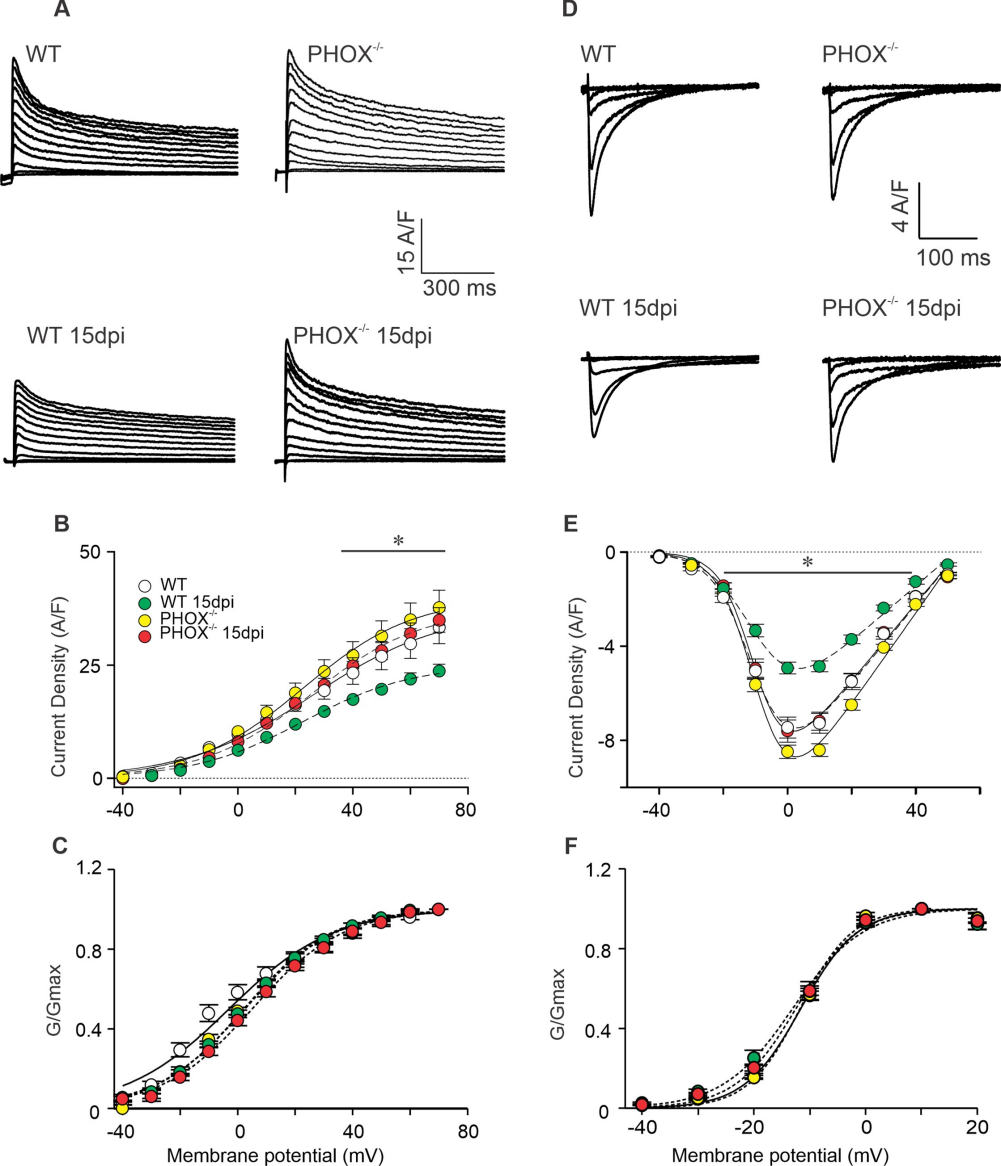
I_Ca-L_ and I_K_ reduction in peak current density during acute phase of chagasic cardiomyopathy is prevented in PHOX^-/-^ mice (A) Representative I_K_ WT (n = 23); WT 15 days post infection (dpi) (n = 23); PHOX^-/-^ (n = 14) and PHOX^-/-^ 15 dpi (n = 16) and ICa-L (D) traces WT (n = 25); WT 15 days post infection (dpi) (n = 25); PHOX^-/-^ (n = 26) and PHOX^-/-^ 15 dpi (n = 19) recorded from experimental groups. Peak current density from I_K_ (B) and I_Ca-L_ (E) were averaged and plotted against membrane potential. Maximum conductance (Gmax) calculated from current-voltage relationship used to normalize the conductance (G) calculated from each tested potential (C and F). No difference in the voltage dependence for channel activation was observed for I_K_ (C) and I_Ca-L_ (F). *p<0.05, compared to WT. Data were compared using One way ANOVA’ test followed by Tukey’s posttest dpi: days post infection. n represents the number of cardiomyocytes.

## References

[1] Santos-Miranda A, Joviano-Santos JV, Ribeiro GA, Botelho AFM, Rocha P, Vieira LQ, et al (2020) Reactive oxygen species and nitric oxide imbalances lead to in vivo and in vitro arrhythmogenic phenotype in acute phase of experimental Chagas disease. PLoS Pathog 16(3): e1008379 10.1371/journal.ppat.1008379 32160269 PMC7089563

