# Supplementary figures and images for: Reactive oxygen species and nitric oxide imbalances lead to in vivo and in vitro arrhythmogenic phenotype in acute phase of experimental Chagas disease
Source: PLoS Pathog. 2020 Mar 11;16(3):e1008379. doi: 10.1371/journal.ppat.1008379 (PMC7089563; doi:10.1371/journal.ppat.1008379)

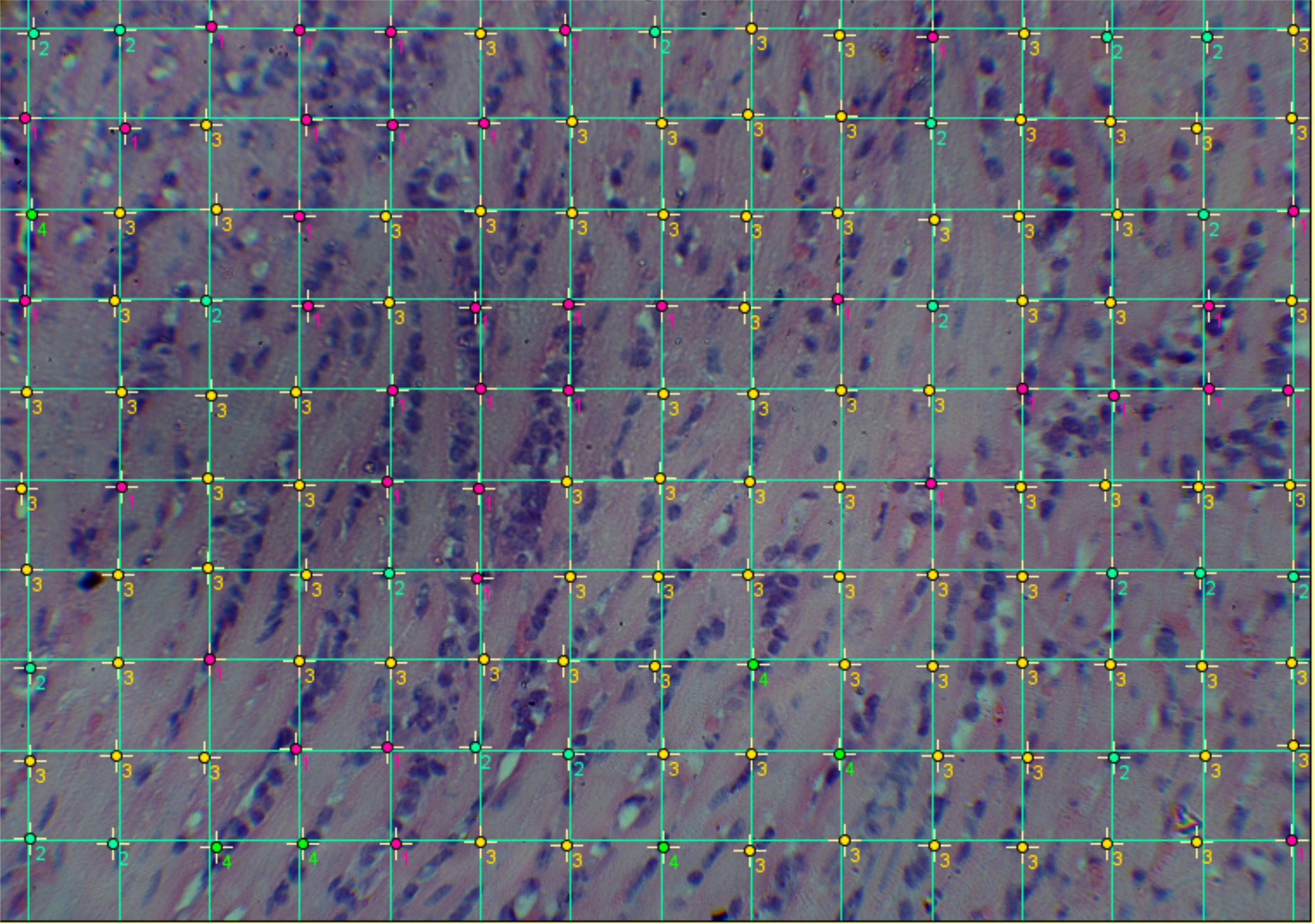

Supplement: S1 Fig — Cardiomyocyte occupancy was calculated by the sum of cardiomyocyte nuclei total number and cardiomyocyte fiber total number. The final values were expressed in percentage. A total of 1,000 grid intersection points were analyzed in different sections per animal. The ImageJ software was used for the grid construction and analysis. (TIF) [file ppat.1008379.s001.tif]

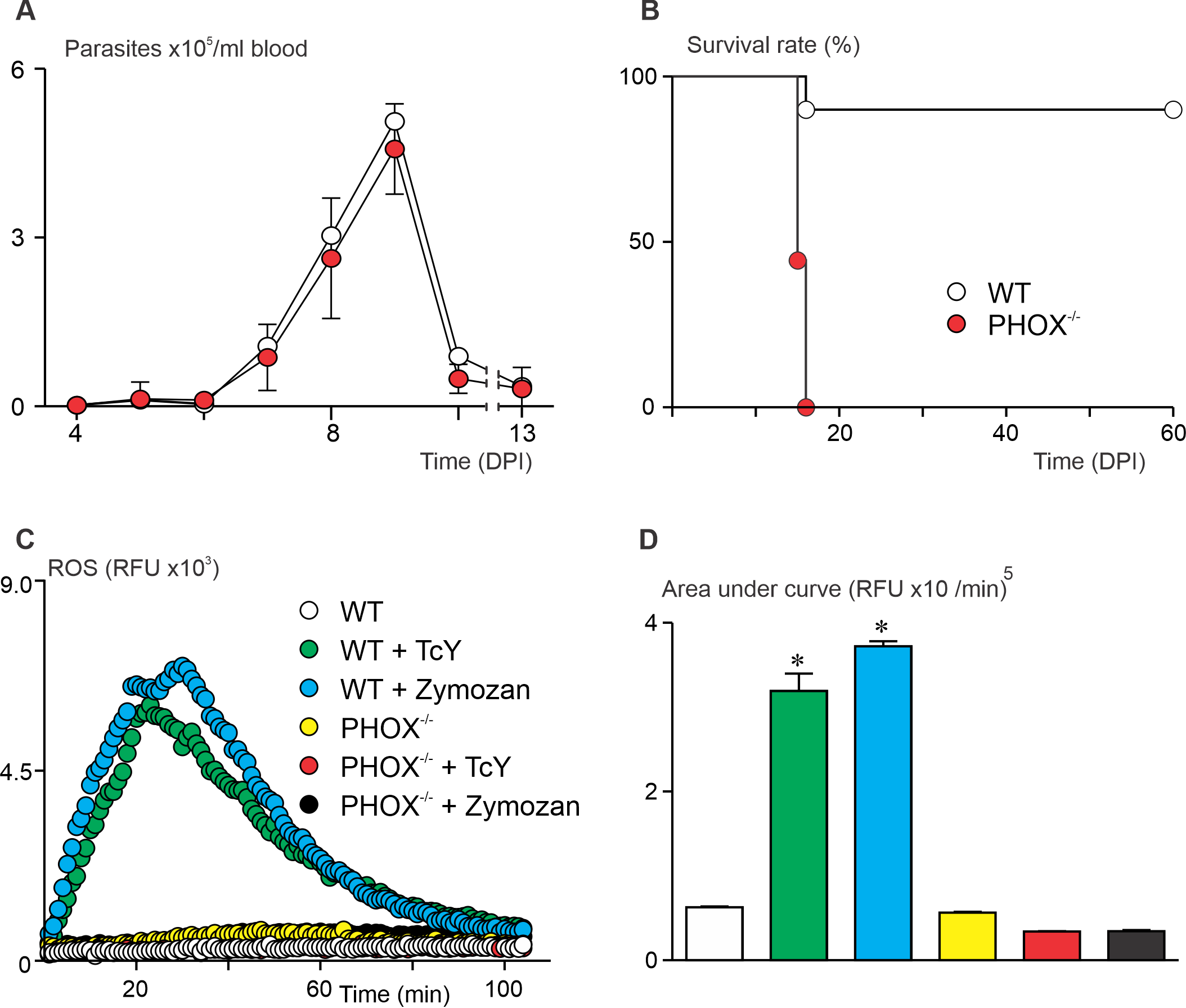

Supplement: S2 Fig — Mice were infected with 1000 blood-born trypomastigotes of Y strain of T. cruzi. Parasitemia (A) and mortality (B) were accessed daily. (A) Points represent mean ± SE of five animals per group from three different experimental infections. (B) Mortality curve is pooled from three experimental infections. C-D: Production of reactive oxygen species by macrophages stimulated with T. cruzi. Macrophages were incubated with 0.5 mM of luminol in culture medium and exposed to T. cruzi trypomastigotes or zymosan. Chemiluminescence was measured immediately and every 2 min, for 120 min (C) The area under curves, representing total ROS production over time was calculated (D) and plotted as mean± S.D. Graphs are representative of three independent experiments performed in triplicate (cells were pooled from three mice for each replicate). * refers to significant differences from the infected and zymosan treated to non-treated macrophages. Data were compared using 2-way ANOVA followed by Bonferroni post hoc test (A-B) or one way ANOVA followed by tukey’s post hoc test (C-D) *p<0.05, compared to WT. RFU: Relative fluorescence units. (TIF) [file ppat.1008379.s002.tif]

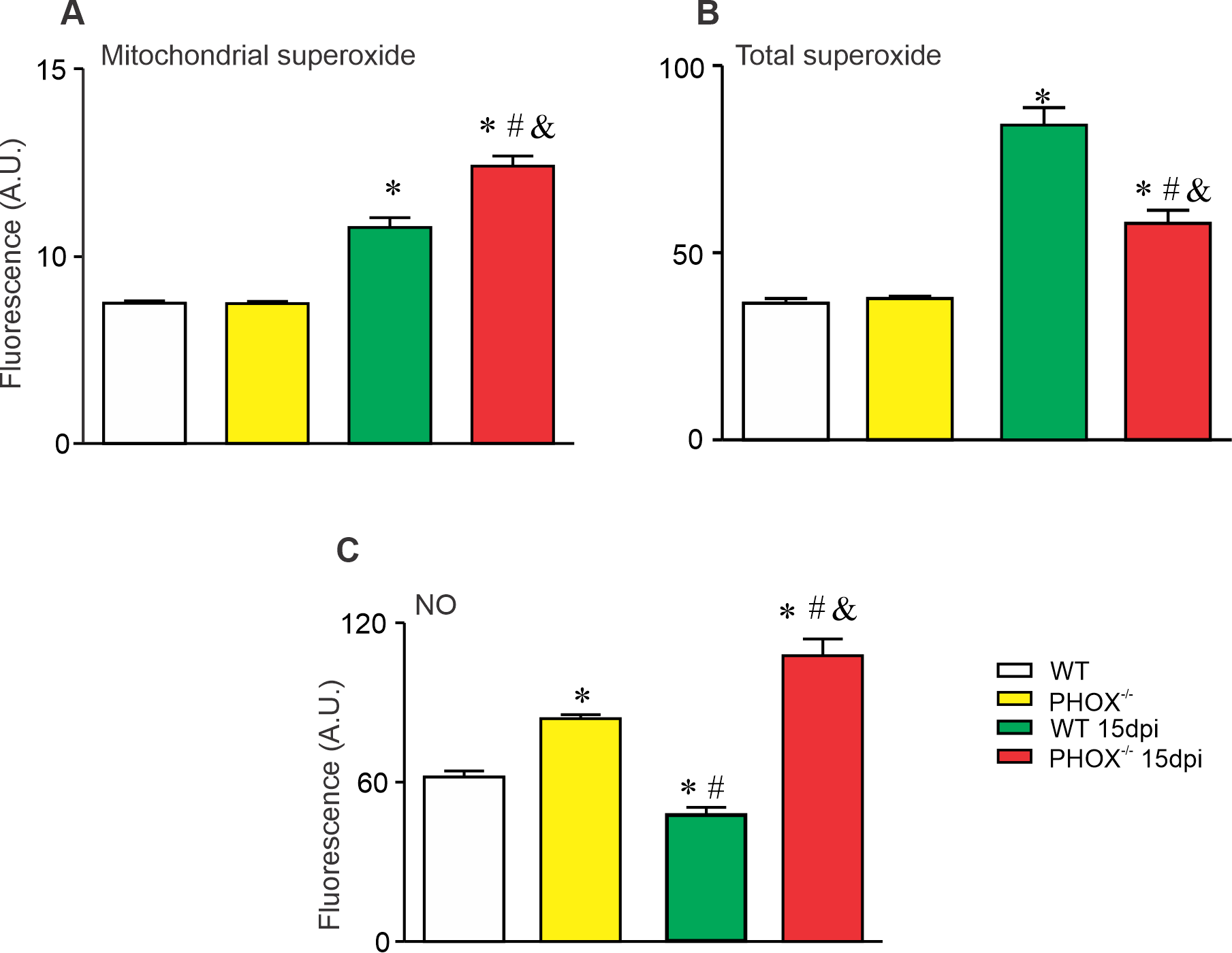

Supplement: S3 Fig — (A) Mitochondrial superoxide production was accessed using 5 μM of MitoSOX probe. WT (n = 94); WT 15 days post infection (dpi) (n = 48); PHOX-/- (n = 100) and PHOX-/- 15 dpi (n = 108). (B) Total production of superoxide, accessed with dihydroethidium probe 5 μM WT, (n = 121); WT 15 dpi, (n = 60); PHOX-/-, (n = 107) and PHOX-/- 15 dpi, (n = 66). (C) NO production, accessed with DAF-FM 5 μM: WT, (n = 94); WT 15 dpi (n = 82); PHOX-/- (n = 112); and PHOX-/- 15 dpi, (n = 117). *p<0.05, compared to WT; #p<0.05, compared to PHOX-/-; &p<0.05, compared to WT 15 dpi. Data were compared using Kruskal-Wallis’ test followed by Dunns’s posttest and plotted as fluorescence arbitrary units (A.U). n represents the number of cardiomyocytes. (TIF) [file ppat.1008379.s003.tif]
